# GOHBA: Improved Honey Badger Algorithm for Global Optimization

**DOI:** 10.3390/biomimetics10020092

**Published:** 2025-02-06

**Authors:** Yourui Huang, Sen Lu, Quanzeng Liu, Tao Han, Tingting Li

**Affiliations:** 1 School of Electrical & Information Engineering, Anhui University of Science and Technology, Huainan 232001, China; huangyourui@ahpu.edu.cn (Y.H.); 2023200770@aust.edu.cn (S.L.); than@aust.edu.cn (T.H.); 15055075771@163.com (T.L.); 2 Anhui Polytechnic University, Wuhu 232000, China

**Keywords:** honey badger algorithm, path planning, golden sine, local optimum

## Abstract

Aiming at the problem that the honey badger algorithm easily falls into local convergence, insufficient global search ability, and low convergence speed, this paper proposes a global optimization honey badger algorithm (Global Optimization HBA) (GOHBA), which improves the search ability of the population, with better ability to jump out of the local optimum, faster convergence speed, and better stability. The introduction of Tent chaotic mapping initialization enhances the population diversity and initializes the population quality of the HBA. Replacing the density factor enhances the search range of the algorithm in the entire solution space and avoids premature convergence to a local optimum. The addition of the golden sine strategy enhances the global search capability of the HBA and accelerates the convergence speed. Compared with seven algorithms, the GOHBA achieves the optimal mean value on 14 of the 23 tested functions. On two real-world engineering design problems, the GOHBA was optimal. On three path planning problems, the GOHBA had higher accuracy and faster convergence. The above experimental results show that the performance of the GOHBA is indeed excellent.

## 1. Introduction

As society continues to evolve, numerous domains, including machine learning [1] and image processing [2], have encountered increasingly intricate optimization challenges over the past few decades. The complexity of these issues arises from the growing volume of data, the multiplicity of practical requirements, and escalating expectations for efficiency and resource optimization [3]. At its core, optimization involves identifying the most favorable solution from the entire spectrum of possible values within a given system.

Conventional optimization techniques, such as the steepest descent method [4] and simplex method [5], are well-suited for straightforward optimization problems because of their more comprehensive algorithmic frameworks and reduced computational demands. Conversely, meta-heuristic algorithms are gaining popularity for tackling various complex problems due to their high solution precision and rapid optimization capabilities. Additionally, meta-heuristic algorithms are straightforward in concept, highly exploratory and exploitative, exhibit minimal dependency on specific problems, and can address more intricate optimization issues. These algorithms draw inspiration from natural, societal, and human behaviors, employing probabilistic search strategies to identify the most optimal solution within the search space [6,7].

Optimization algorithms are crucial in various fields. In engineering, optimization algorithms can improve the performance and cost-effectiveness of mechanical designs, electronic layouts, and civil structures. In economics, optimization algorithms optimize financial portfolios and supply chain operations. In computer science, they improve algorithmic efficiency and machine learning models. In medicine, they help with medical imaging and drug development, and optimization algorithms provide important support for advances in all fields.

In the past few years, scholars have proposed new algorithms, improved strategies, and applied meta-heuristics to practical problems in various fields. Ahmed et al. applied the Whale Optimization Algorithm (WOA) to the cost and emission scheduling of thermal power plants in energy hubs under multi-objective scenarios [8]. Farzad Kiani et al. for the complex constrained optimization problem proposed a new hybrid meta-heuristic chaotic sand cat swarm optimization algorithm, which incorporates chaotic features of non-repeating locations into SCSO to improve the global search performance and convergence behavior [9]. Jin Wu and Zhendong Su proposed a new algorithm called the Flavoring Search Algorithm (FSA). Inspired by human flavoring behavior, it balances exploration and exploitation by introducing a unique flavoring factor, which significantly improves the global search performance and convergence behavior of the algorithm, especially in solving multimodal functions and complex engineering optimization problems [10]. YaShen et al. proposed a variant of the WOA based on multiple swarm evolution (MEWOA), which solves the problem of the Whale Optimization Algorithm (WOA) that is slow to converge and prone to fall into local optimum [11].The optimization problems including the single-peak function, multi-peak function, fixed-dimension multi-peak function, CEC 2019 test suite, and CEC 2014 test suite were solved by simulating the behavior of hippopotamuses, including position update, defense strategy, and predator avoidance [12]. This was proposed by Hernán Peraza-Vázquez et al. and inspired by the hiding, skin darkening or brightening, blood spraying, and move-and-run defense behaviors of horned lizards for solving optimization problems [13]. Although meta-heuristic algorithms are very effective in solving complex optimization problems, they have some general challenges, such as the possibility of falling into local optima, and the performance of the algorithms may suffer as the problem’s dimensionality increases.

The honey badger algorithm (HBA) [14] has tremendous advantages in optimization problems. Yuefeng Xu et al. proposed a Symbiosis-based Honey Badger Algorithm (SHBA), which integrates information exchange between two different populations by modeling the cooperative symbiosis mechanism between honey badgers and honeycreepers, and employs multiple strategies to increase population diversity while maintaining efficient search performance [15]. Parijata Majumdar and Sanjoy Mitra proposed an enhanced honey badger algorithm (NGS-eHBA) based on nonlinear adaptive weights and a golden sine operator. The newly added nonlinear adaptive weights are able to adaptively explore the search space, balancing its diversity and enhancement [16]. Junjie Sun et al. significantly improved the prediction accuracy, stability, and computational efficiency of the algorithm by introducing improvements such as good point set initialization, classification parameter value tuning, a chaotic sequence density factor, deep belief network optimization, chaos theory, and a sigmoid-based acceleration factor [17]. Rajendran Arul Jose et al. proposed a hybrid technique to optimize the steady-state currents within a smart grid (SG) by combining a DC/DC converter with a DC/AC inverter. The proposed method combines the Gradient Boosted Decision Tree (GBDT) and honey badger algorithm (HBA), often referred to as the GBDT-HBA method [18]. Lei Guo initialized the population by introducing a set of good points, categorically adjusted the values of the parameter di, improved the density factor by applying chaotic sequences, and optimized the feature extraction combined with the Variable Difference Mode Decomposition (VMD) in terms of accuracy and stability in the prediction of wind power, achieving a significant improvement [19]. Timur Düzenli et al. aimed to improve the convergence performance of PV systems by introducing a chaotic HBA with Gaussian/mouse mapping and a hybrid approach based on dyadic learning and the HBA [20]. From the above references, we can learn that the HBA has the advantages of simple implementation, global search capability, and wide adaptability, but it also has the disadvantages of premature convergence, slower convergence, sensitivity to parameter selection, and possible loss of diversity. Many experimentalists have made improvements. Zhiwei Ye, Tao Zhao et al. improved the stochastic control parameters by introducing Tent chaotic mapping and composite variational factors and proposed an HBA (IHBA) improved by fusing multiple strategies [21]. Bo Yang et al. significantly improved the global search capability by introducing good point set initialization, improved parameter classification, chaotic sequence density factor updating, variational pattern decomposition, and other improvement measures, which significantly enhanced the feature extraction capability and prediction accuracy of wave energy data, as well as the stability and computational efficiency of the algorithm [22]. Ajay Kunmar Bansal et al. proposed an HBA algorithm for HNGS optimization and demonstrated its superiority in solving global optimization problems with multiple local minima [23]. Peixin Huang et al. proposed an improved HBA (ODEHBA) in combination with other optimization strategies and adaptive tuning mechanisms to enhance its search efficiency and global exploration in complex search spaces [24]. Oluwatayomi Rereloluwa Adegboye et al. proposed a new HNGS optimization algorithm based on the golden sine (GS) mechanism of the honey badger algorithm (HBA) with the Tent Chaos (TC) obtained. TC was obtained as the new optimization algorithm (GST-HBA). The main goal of this algorithm is to balance exploration and exploitation more efficiently in the optimization process, leading to fast convergence and overall diversity [25]. Although the above studies can improve the performance of the HBA, continued improvement and optimization are still important, and there is a need to further enhance the convergence speed of the HBA as well as its ability to jump out of the local optimum and enhance the diversity of species to cope with complex optimization problems.

The objective function and constraint types are defined in the field of engineering optimization and in quadruped robot path planning. In robot path planning, the objective is to minimize the total path length from the start point to the end point, ensure that the path is collision-free and continuous, and constrain map boundaries and obstacle avoidance. In the robot gripper problem, the objective is to optimize the gripping force and energy efficiency while adhering to constraints such as force limitations, accuracy, and physical dimensions to ensure safe and efficient operation. With respect to the gearbox problem, the focus is on minimizing the size of the gearbox while maintaining a specified level of efficiency, with constraints on torque capacity, output speed, and material strength to ensure performance and durability. These optimization issues are critical to improving the efficiency and effectiveness of robotic systems and mechanical components and drive innovation in design and operation.

In order to solve the above problems and further improve the ability of HBA to address optimization questionsbe, in this paper, a GOHBA is proposed, which firstly introduces Tent chaotic mapping initialization to enhance the population diversity and initialized population quality of the HBA. In addition, replacing it with a new density factor enhances the ability to handle complex optimization problems with multiple local optimal solutions. Finally, the introduction of the golden sine strategy enhances the HBA’s ability to search the problem space, effectively increasing its ability to jump out of the local optimum. The main contributions of this paper are as follows:

(1) The introduction of the Tent Chaos algorithm for initialization improves the diversity of the population and the quality of the initial population to achieve better optimization results.
(2) The use of a new density factor helps the algorithm to explore more extensively in the whole solution space, especially in the early stage of the algorithm, which can effectively avoid premature convergence to a local optimal solution.
(3) The golden sine strategy is introduced to improve the global search capability, accelerate the convergence speed, and help avoid falling into local optimal solutions.
(4) Test the GOHBA on 23 test functions. Successfully solve two examples of engineering optimization problems as well as a quadruped robot path planning problem.

Section Plan:

Section 2: introduces the honey badger algorithm and proposes an improved GOHBA.
Section 3: Compares the GOHBA with seven algorithms using 23 test functions. Analyzes performance via statistical tests and convergence analysis.
Section 4: demonstrates the GOHBA’s application in engineering optimization and quadruped robot path planning.
Section 5: summarizes experimental results, discusses limitations, and explores future development directions, such as integrating the GOHBA with other techniques.

## 2. Algorithm Analysis

The honey badger algorithm (HBA) is a new type of intelligent optimization algorithm, proposed by Fatma A. Hashima et al. in 2021, inspired by the honey badger’s hunting behavior in nature, which mainly seeks for optimization by simulating the honey badger’s intelligent foraging behavior, and has the characteristics of strong optimization ability and fast convergence speed.

### 2.1. The Honey Badger Algorithm

The initialization phase involves initializing the number of honey badgers (population size N), and their respective random positions. These positions are determined by Equation (1):

(1) $$x_{i}=lb_{i}+r_{i}\times \left(ub_{i}-lb_{i}\right)$$

where $r_{i}$ is a random number between 0 and 1, $x_{i}$ is the position of the ith honey badger position of a candidate solution from one of the N populations, $lb_{i}$ and $ub_{i}$ are the lower and upper bounds of the search domain, respectively. The intensity is related to the concentration of the prey and the distance between it and the prey. The intensity of the prey’s odor $I_{i}$, be shown in Equation (2), follows the inverse square law: if the odor is high, the movement will be fast and vice versa.

$$I_{i}=r_{2}\times \frac{S_{i}}{4\pi {d_{i}}^{2}}$$

(2) $$S={\left(x_{i}-x_{i+1}\right)}^{2}$$

$$d_{i}=x_{prey}-x_{i}$$

where $r_{2}$ is a random number between 0 and 1; source strength *S* denotes the strength of the prey’s odor; the higher the value, the more acutely the honey badger perceives the prey, and it approaches quickly. Calculations were made with individual positions i ranging from 1 to 29, with the last honey badger having a source strength of ${\left(x_{30}-x_{1}\right)}^{2}$ to ensure that it did not exceed the population size (30); $d_{i}$ is the distance between the prey and the ith badger; $x_{prey}$ is the location of the hive, which is treated as the location of the optimal individual in the algorithm. The closer the honey badger is to the hive, the stronger this attraction is.

The density factor (α) controls the time-varying randomization and ensures a smooth transition from exploration to exploitation. As the number of iterations increases, the density factor decreases and the expression is shown in Equation (3):

(3) $$\alpha =C\times \exp(\frac{-t}{t_{\max}})$$

where t is the current iteration number, $t_{\max}$ is the maximum number of iterations, C=2.

This step and the next two steps are used to escape the local optimal solution region. In this case, the HBA uses a flag F to change the search direction to take advantage of higher search efficiency agents strictly scanning the search space.

As mentioned before, the HBA position update process ($x_{i}^{new}$) is divided into two parts: the “digging phase” and the “honey phase”. A better explanation is given below.

In the digging phase, the action of the honey badger can be modeled by Equation (4):

(4) $$x_{i}^{new}=x_{prey}+F\times \beta \times I\times x_{prey}+F\times r_{3}\times \alpha \times d_{i}\times |\cos(2\pi r_{4})\times [1-\cos(2\pi r_{5})]|$$

where $x_{prey}$ is both the location of the prey and also the best location found so far, i.e., the global best location. β ≥1 (default = 6) is the ability of the honey badger to acquire food. $d_{i}$ is the distance between the prey and the ith honey badger. $r_{3}$, $r_{4}$, and $r_{5}$ are three different random numbers between 0 and 1. F serves as a flag to change the search direction and is determined by $r_{6}$; *F* is 1 if $r_{6}$ ≤ 0.5 and −1 otherwise ($r_{6}$ is a random number between 0 and 1). During the digging phase, honey badgers rely heavily on the odor intensity I of the prey $x_{prey}$, the distance $d_{i}$ between the badger and the prey, and the time-varying search influence factor α. In addition, during digging activities, the badger may be exposed to any disturbance, which allows it to find better prey locations.

A honey badger following a honeyguide bird to the hive can be modeled by equation (5):

(5) $$x_{i}^{new}=x_{prey}+F\times r_{7}\times \alpha \times d_{i}$$

where $x_{i}^{new}$ refer to the new position of honey badger, $x_{prey}$ is the prey position, F and α are determined by the random number $r_{6}$ and Equation (3), and $r_{7}$ is a random number between 0 and 1. From Equation (5), the honey badger searches near the prey position x based on the distance information $d_{i}$. At this stage, the search is affected by the time-varying search behavior (α). In addition, the honey badger may find the disturbance F.

### 2.2. The Proposed Algorithm

#### 2.2.1. Tent Sequence Initialization Population

In traditional HBAs, the initial population is often randomly generated, so it is easy to have a poor-quality population. Poor initial population quality will weaken the search efficiency of the honey badger algorithm, increase the risk of premature convergence, reduce population diversity, and affect the convergence speed and accuracy of the algorithm. Chaotic mapping has the characteristics of randomness, irreducibility, and chaotic traversal, which can generate uniformly distributed populations, making it an important application in the design of optimization algorithms. Therefore, Tent Chaos [26] is introduced in this paper for population initialization, and the reason for choosing Tent Chaos is that it can generate Gaussian-distributed random numbers with better stochasticity, which has a certain exploration ability. The method of initializing the population using Tent Chaos can improve the diversity of the population and the quality of the initial population, thus achieving better optimization results.

The expression of the Tent mapping structure is shown in (6):

(6) $$x_{n+1}=\left\{\begin{array}{l} \frac{x_{n}}{a},0\leq x_{n}\leq a\\ \frac{\left(1-x_{n}\right)}{1-a},a<x_{n}\leq 1 \end{array}\right.$$

where 0 < *a* < 1. Generally, *a* is taken as 0.5.

After generating chaotic sequences using Tent chaotic mapping, the chaotic sequences are mapped to the search space of the optimization problem, and then the mapped values are used as the individuals of the initial population, and the initialization formula is used to complete the initialization of the population, so as to improve the diversity and uniform distribution of the initial population, enhance the global search ability and convergence speed of the algorithm, and make the honey badger algorithm more efficient and stable in solving complex optimization problems.

**Figure 1:**
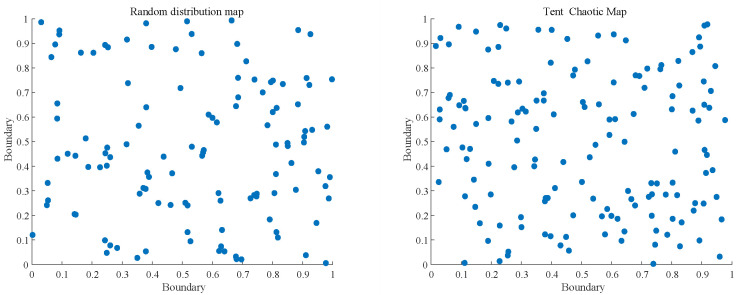
Distribution of random initialization, Tent mapping initialization.

As shown in Figure 1, the first figure shows the random initialization and the second figure shows the Tent mapping initialization. As can be seen from the figure, the Tent mapping introduced in this paper has greatly improved the uniformity of distribution. Therefore, Tent mapping can be applied to the initialization to improve the initial mass difference of the population.

#### 2.2.2. Introduction of New Density Factors

In the HBA, the density factor plays a key role in controlling the time-varying randomization of the search process and ensuring a smooth transition from the exploration phase to the exploitation phase. The algorithm sets the initial value of the density factor to 2 and lets it decrease to 0 as the number of iterations increases, a process that follows Equation (3). However, the approximate linear decreasing approach may make the algorithm prone to falling into local optimal solutions that are difficult to escape from, thus affecting the convergence accuracy of the algorithm, especially when dealing with complex and high-dimensional problems. This is because the algorithm may not be able to fully cover and explore the entire solution space, resulting in limited convergence accuracy. Therefore, choosing an appropriate density factor is crucial for the performance of the algorithm.

The introduction of new variables helps to enhance the searching ability of the algorithm: the introduction of the new variable α increases the complexity of the searching process and improves the searching ability, as shown in Equation (7). Other algorithms have proposed different density factors: study [27] proposes a five-fold nonlinear density factor that enhances the search ability of the algorithm, as shown in Equation (8). Study [28] proposes a density factor based on sinusoidal functions, which facilitates the search for the optimal value in the global range, as shown in Equation (10).

**Figure 2:**
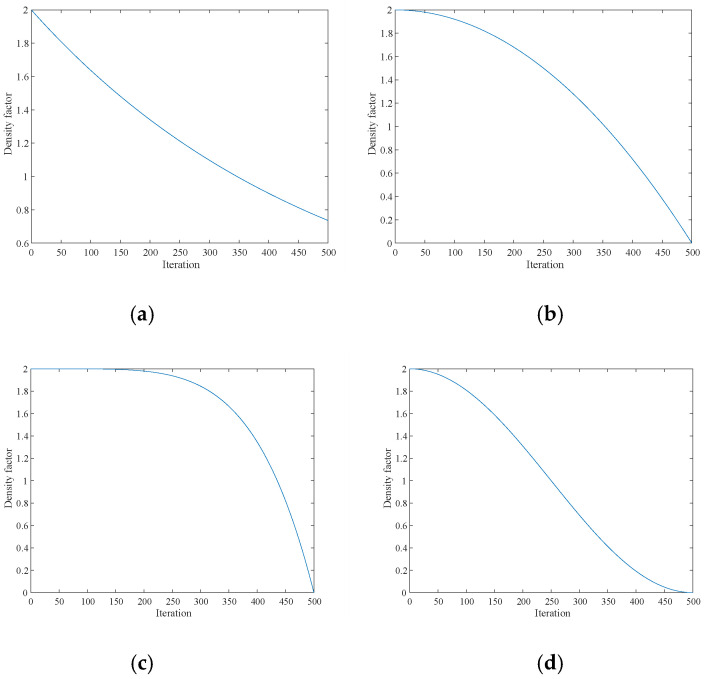
(**a**) The original density factor in Equation (3); (**b**) the new density factor in Equation (7); (**c**) the quintic nonlinear density factor in Equation (8); and (**d**) the sinusoidal function density factor in Equation (9).

Figure 2 illustrates the above four density factors, where (a) is the original density factor of Equation (3), (b) is the nonlinear density factor of Equation (7), (c) is the nonlinear density factor of five times as demonstrated in Equation (8), and (d) is the density factor proposed in Equation (9).

(7) $$\alpha =C*(1-{\left(iter_{C}/iter_{\max}\right)}^{2})$$

(8) $$\alpha =C-C*{\left(iter_{C}/iter_{\max}\right)}^{5}$$

(9) $$\alpha =1+\sin(\pi /2+\pi *iter_{c}/iter_{\max})$$

where $iter_{C}$ is the current iteration number and $iter_{\max}$ is the maximum iteration number.

When dealing with complex multimodal functions, the HBA often encounters the problem of insufficient accuracy. To solve this problem, this paper introduces a density factor based on the sine function. The periodicity feature of the density factor based on the sine function helps the algorithm to explore more extensively in the whole solution space, especially in the early stage of the algorithm, which can effectively avoid converging to the local optimal solution too early. As the iterations proceed, the dynamically changing nature of the sine function allows the algorithm to gradually narrow down the search range and focus on promising regions for a more detailed search. In addition, the introduction of the sinusoidal function helps the algorithm to adaptively adjust the search step size and direction during the search process, thus increasing the likelihood of finding a globally optimal solution. These properties of the sine function make it particularly suitable for dealing with complex optimization problems with multiple local optimal solutions, enhancing the robustness and reliability of the algorithm.

#### 2.2.3. Gold Sine Strategy

The Golden Sine Algorithm (GSA) [29] originates from the search of a space similar to the solution of the problem to be optimized by scanning within the unit circle of the sine function and shrinking the search space by the golden ratio to approximate the optimal solution of the algorithm.

Like other population-based optimization algorithms, the Gold-SA starts with a randomly generated population. The initial population of the Gold-SA is designed to better scan the search space by randomly generating a uniform distribution in each dimension, whose expression is shown in Equation (1).

Where $x_{i}$ is the initial value of the ith individual and $l_{b}$,$u_{b}$ are the upper and lower limits of the search space.

The GSA introduces golden section coefficients $x_{1}$ and $x_{2}$ in the location updating process to achieve a good balance between “search” and “exploitation”; these coefficients reduce the search space and lead individuals to the optimal value. $x_{1}$, $x_{2}$ expressions are shown in Equations (10) and (11):

(10) $$x_{1}=a\times (1-\tau )+b\times \tau$$

(11) $$x_{2}=a\times (\tau +b\times (1-\tau ))$$

In Gold-SA, initial default values for *a* and *b* are considered to be -π and π, respectively. These two coefficients are applied to the current and target values in the first iteration. Then the coefficients $x_{1}$ and $x_{2}$ are updated as the target value changes, τ for the golden ratio, $\tau =(\sqrt{5}-1)/2$.

As the number of iterations increases, the GSA performs a position update via Equation (12):

(12) $$x_{i}^{t+1}=x_{i}^{t}\times |\sin(r_{1})|-r_{2}\times \sin(r_{1})\times |x_{1}\times D_{i}^{t}-x_{2}\times x_{i}^{t}|$$

where $x_{i}^{t+1}$ is the (t+1)st iteration position of the ith individual; $x_{i}^{t}$ is the tth iteration position of the ith individual; $D_{i}^{t}$ is the tth iteration optimal position of the ith individual; $r_{1}$ is a random number in the range of [0,2π]; $r_{2}$ is a random number in the range of [0,π]; and $x_{1}$, $x_{2}$ are the golden section coefficients.

The Golden Sine Strategy (GSS) plays a role when the search space becomes gradually narrower and searches in the region where the optimal solution may be reached in order to improve the convergence efficiency. The introduction of the Golden Sine Strategy enables the improved GOHBA to better balance the global and local searches during the search process and improve the optimization efficiency.

#### 2.2.4. Algorithm Flow

**Figure 3:**
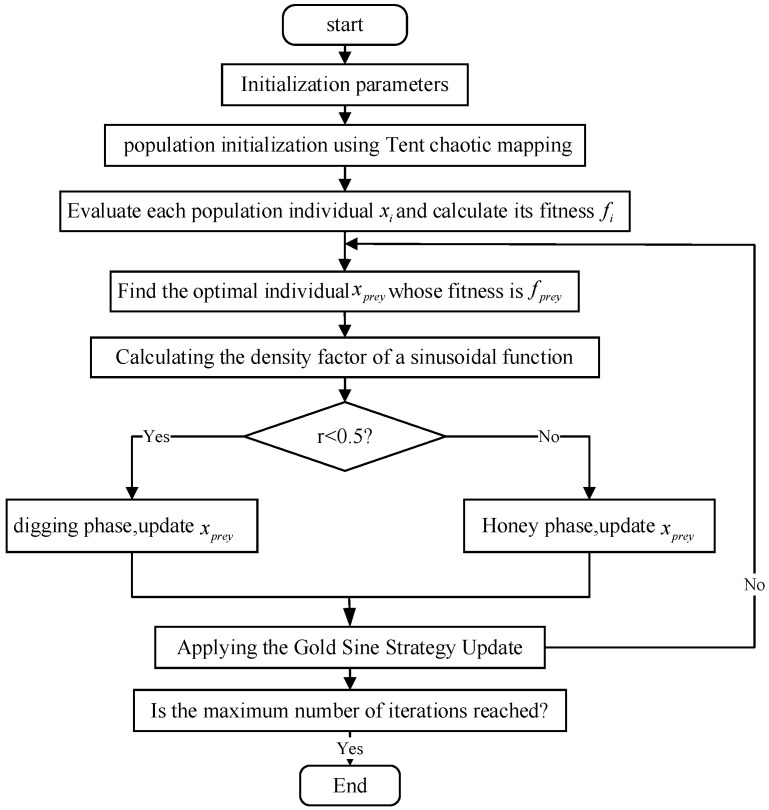
Flowchart of the global optimization HBA.

The GOHBA calculates the density factor by initializing the parameters and population, then assigning an initial position to each individual using the Tent sequence, followed by evaluating their fitness and using a convergence factor based on a sinusoidal function. The core of the algorithm lies in the sinusoidal gold strategy, which guides individuals to explore new solutions or perform a local search for the current solution during the digging and nectar harvesting phases, as well as checking whether the new solution is out of bounds and updating the individual position and fitness. After each round of iteration, the algorithm updates the global optimal solution until the termination condition is satisfied, and finally outputs the optimal solution. The flowchart of the algorithm is shown in Figure 3.

### 2.3. Complexity Analysis

#### 2.3.1. Computational Complexity

Big O notation (BOT) [30] is a mathematical tool used in computer science to describe the complexity of an algorithm. It is used to define the tendency of an algorithm to grow in runtime or require storage space as the input size increases.

The computational complexity of the control parameters defined by the HBA is O (P × N), where P denotes the overall size and N denotes the problem size. The time required in the initialization phase of the algorithm is also O (P × N). Meanwhile, the computational complexity of updating the agent locations is also O (P × N). It is obvious from the pseudocode of the GSCSO algorithm that the computational cost of the initialization phase is O (P × N), while the complexity of the GSCSO algorithm to evaluate the fitness of each individual during the iteration process is O (T × P × N), where T represents the number of iterations. With the introduction of the Golden Sine Strategy, the computational complexity becomes O (2 × T × P × N). Taken together, the overall computational complexity of the GOHBA is O (T × P × N), which is the same as the original HBA.

#### 2.3.2. Space Complexity

Space complexity is a theoretical computer science metric used to quantify the efficiency of an algorithm in terms of memory usage, reflecting the storage resources required to run the algorithm. In meta-heuristic algorithms, space complexity is mainly affected by the population size and problem dimension. Specifically, the space complexity of the HBA is defined as O (P × N) when the population size of the algorithm is P and the problem dimension handled is N. The GOHBA also employs the same size of population and the same problem dimension, and thus, its space complexity is also O (P × N). Taken together, the HBA and GOHBA do not differ in space complexity; the GOHBA does not add additional storage requirements to improve performance.

### 2.4. The Path Planning Optimization Problem

**Figure 4:**
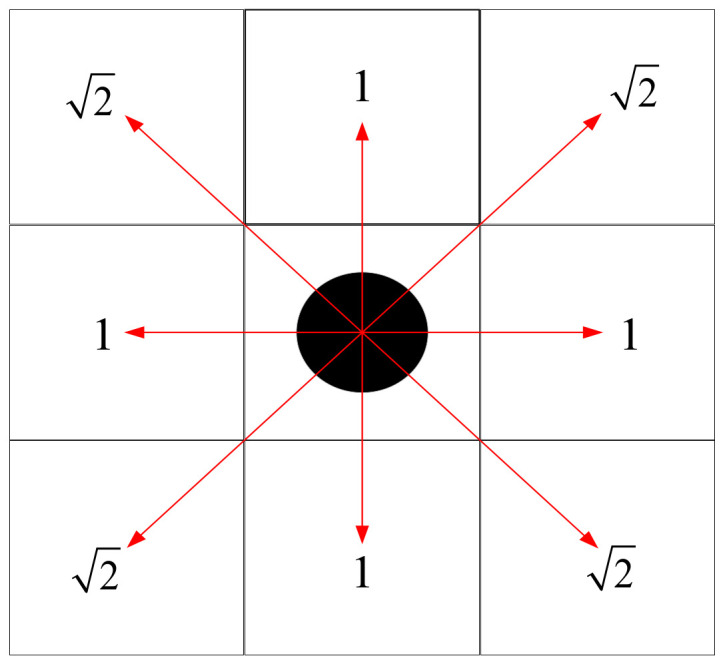
Feasible area of the robot.

In the pursuit of robot path planning research, the goal is to determine the most efficient route that enables the robot to navigate seamlessly from its initial position to the intended destination. To this end, a grid map has been developed to emulate the robot’s movement environment, where each grid cell signifies a distinct spatial segment. In this model, cells occupied by obstacles are marked with a 1, while those that are clear for the robot’s movement are marked with a 0. Each grid cell is surrounded by eight adjacent cells, which could be potential subsequent positions for the robot’s path. The distance between adjacent cells is calculated using Euclidean distance, which is either 1 or $\sqrt{2}$, contingent on their positioning, as depicted in Figure 4. In this illustration, the black dot signifies the robot’s current location and the red arrows point to the directions available for the robot’s movement.

The transformation relationship between the grid sequence number and the corresponding coordinates is shown in (13) [31]:

(13) $$\left\{\begin{array}{l} x_{n}=\mathrm{mod}(n,R_{x})-0.5\\ y_{n}=R_{y}+0.5-ceil(n/R_{y}) \end{array}\right.$$

where $\left(x_{n},y_{n}\right)$ denotes the position coordinates of the nth grid, $x_{n}$ represents the horizontal and vertical coordinates of the nth grid, $y_{n}$ denotes the vertical coordinates of the nth grid, $R_{x}$ denotes the total number of rows of the environment model, $R_{y}$ denotes the totalnumber of columns of the environment model, ceil the number of the nth grid,ceil() and mod () are the round-down function and remainder function, respectively.

After setting up relevant environmental data, a population intelligence algorithm is used to find the ideal path in the map that satisfies all requirements. In addition, a fitness function that can contain constraints is created, and solutions that can satisfy this function are retained. Those that do not satisfy the fitness function are eliminated.

The robot’s moving path must be confined within the boundary of the raster map, and the constraints lb and ub are the boundaries of the search space for path planning. Any node $\left(x_{i},y_{i}\right)$ in the robot’s moving path must satisfy the following boundary conditions:

(14) $$\left\{\begin{array}{l} lb_{x}\leq x_{i}\leq ub_{x}\\ lb_{y}\leq y_{i}\leq ub_{y} \end{array}\right.$$

where $lb_{x}$ and $ub_{x}$ are the lower and upper limits of the horizontal boundary, $lb_{y}$ and $ub_{y}$ are the lower and upper limits of the vertical boundary, and i belongs to any value.

The robot’s movement path in the access area should avoid overlapping paths and detours. Assuming that the position coordinate of the robot at the moment i is $\left(x_{i},y_{i}\right)$, the position coordinate of the robot at the next moment $\left(x_{i+1},y_{i+1}\right)$ should be satisfied:

(15) $$x_{i+1}>x_{i}\;\mathrm{or}\;y_{i+1}>y_{i}$$

We use $S_{o}$ to represent all grids occupied by obstacles, and $S_{g}$ to represent all reachable target grids. The core of the path planning problem is to find an optimal path from the starting point to the target point in the set. Suppose the robot starts from the starting point and passes through H nodes. Our objective function is to minimize the total distance L traveled by the robot on the unobstructed path, as shown in Equation (16). By optimizing this objective function, we can plan a path for the robot that is both efficient and safe.

(16) $$\min L=\sum_{i=1}^{H}\sqrt{{\left(x_{i+1}-x_{i}\right)}^{2}+{\left(y_{i+1}-y_{i}\right)}^{2}}$$

In Equation (16), the positions of the robot before and after the movement are $\left(x_{i},y_{i}\right)$ and $\left(x_{i+1},y_{i+1}\right)$. The smaller the value of the total moving distance L is, the shorter the path is. In order to achieve path optimization, the planning objective is to minimize the path length, and finally, the optimal path that satisfies all the constraints is found by minimizing the path length.

## 3. Experiments

### 3.1. Experimental Setup and Assessment Criteria

The experimental environment for the improved GOHBA, and the other algorithms proposed in this paper is Windows 10. The processor is AMD Ryzen 9-7940H w/ Radeon 780M Graphics 4.00 GHz and 16G operating memory. The simulation implementation is realized through MATLAB R2023a.

We use 2 test functions to test the performance of the HBA and its improved algorithms, using the following evaluation criteria for each algorithm.

Mean value: the mean value is the average value calculated after the algorithm executes the test function in multiple cycles. The formula is shown in Equation (17):

(17) $$Mean=\frac{1}{S}\sum_{i=1}^{S}F_{i}$$

where S is the number of cycles, *F_i_* is the result of the ith independent experiment.

Std: the standard deviation is the standard deviation calculated after the algorithm loops through multiple executions of the test function. The calculation formula is shown in Equation (18):

(18) $$Std=\sqrt{\frac{1}{S}\sum_{i=1}^{S}{\left(F_{i}-\frac{1}{S}\sum_{i=1}^{S}F_{i}\right)}^{2}}$$

where S is the number of cycles and *F_i_* is the result of the ith independent experiment.

### 3.2. Test Functions

In order to validate the performance of the HBA improvements proposed in this paper, 23 standard benchmark test functions were selected for testing. Among them, the unimodal function Unimodal ($F_{1}\sim F_{7}$) has only one global optimum and is therefore used to evaluate the development capability of the optimization method. In contrast, the multimodal function Multimodal ($F_{8}\sim F_{13}$), which has multiple local optimal regions, tests optimization methods capable of eliminating local optima and finding the global optimal location. Fixed-dimension test function Fixed-dimension ($F_{14}\sim F_{23}$) has numerous local optima, so the fixed-dimension multimodal function is used in meta-heuristic algorithms to evaluate the exploration and exploitation balance. The detailed description is shown in Table 1, where D and R represent the dimensionality of the function, the boundary of the search space, and the objective function value at the optimal location, respectively.

### 3.3. Sensitivity Analysis

The results obtained by meta-heuristic algorithms are usually influenced by the number of fitness evaluations FEs=p×t. Most of the literature sets FEs to 15000 (FEs = 30 ∗ 500 = 15000), where the overall size p=30 and the number of iterations is t=500. Different sets of *p* and t lead to performance differences. Therefore, we chose three different p/t sets to analyze their impact on the GOHBA: 15/1000, 30/500, and 60/250, respectively. Twenty-three test functions were selected for sensitivity analysis; the experimental results are shown in Table 2.

As shown in Table 2, the 6 test functions $F_{1}$,$F_{3}$,$F_{9}$,$F_{10}$,$F_{11}$,$F_{17}$ showed the best results on all 3 different p/t sets. The 8 test functions $F_{2}$,$F_{4}$,$F_{7}$,$F_{8}$,$F_{12}$,$F_{19}$,$F_{21}$,$F_{23}$ have the smallest Std and perform the best on p/t of 30/500; the 6 test functions $F_{13}$,$F_{14}$,$F_{15}$,$F_{18}$,$F_{20}$,$F_{22}$ have the smallest Std and perform the best when p/t is 60/250; for the 4 test functions $F_{2}$,$F_{5}$,$F_{6}$,$F_{16}$, Std is the smallest and performs best when p/t is 15/1000. Rank-Count is the sum of the rank values of all the functions for the same set of p/t, in which the Rank-Count value of 40 is smallest when p/t is 30/500. Ave-Rank denotes the average rank value of the 23 test functions. Through the Friedman test, we found that it works best when p/t is 30/500. Therefore, in this paper, we set p/t to 30/500.

### 3.4. Experimental Results

To evaluate the performance of the HBA and its variants (HBA1 and HBA2), we conducted experiments with 23 test functions. In these experiments, one of them, HBA1, replaced the original density factor with Equation (10), while HBA2 was updated with Equation (6) for Tent mapping population initialization and introduced the Golden Sine Strategy. In addition, we compared them with the Black-winged Kite Optimization Algorithm (BKA), the Educated Competitive Optimizer Algorithm (ECO), the Goose Optimization Algorithm (GOOSE), and the Newton–Raphson Optimization Algorithm (NRBO). Table 3 shows the experimental results of these algorithms over 50 cycles.

Based on the data in Table 3, it is possible to see how the GOHBA and its comparison algorithms perform on several test functions. Specifically, GOHBA outperforms the other algorithms in terms of mean and standard deviation on functions $F_{1}$ through $F_{4}$. On functions $F_{5}$ and $F_{6}$, the mean and standard deviation of the GOHBA are slightly inferior to HBA2, but still better than the other algorithms, while on function $F_{7}$, the GOHBA mean is still better than the other algorithms, and is only lower than HBA2 in terms of standard deviation. For the multimodal test functions $F_{8}$ to $F_{13}$, the performance of the GOHBA on the four test functions $F_{8}$,$F_{9}$,$F_{10}$ and $F_{11}$ is also better than that of the other algorithms. For only two functions, $F_{12}$ and $F_{13}$, the mean and standard deviations of the GOHBA are slightly lower than that of HBA2, but still better than the other algorithms. In the fixed dimensional test function $F_{14}$ to $F_{23}$, the GOHBA optimizes the mean on all seven test functions $F_{15}$~$F_{17}$,$F_{19}$,$F_{21}$~$F_{23}$. The standard deviation of the GOHBA is also optimized on the 3 test functions $F_{17}$,$F_{18}$,$F_{22}$, and only on the 3 test functions $F_{15}$,$F_{21}$ and $F_{23}$ is the standard deviation of the GOHBA slightly lower than that of HBA2, but still better than other algorithms.

The combined experimental results conclude that the GOHBA shows a clear advantage on the above test functions, indicating that the GOHBA has better stability, a stronger ability to get rid of local optimization, and overall better performance.

### 3.5. Friedman Calibration

To further compare the overall performance of the eight algorithms, the algorithms can be ranked using the Friedman test [32], and the performance rankings of the eight algorithms, including the GOHBA, with respect to the above 10 benchmark functions are shown in Table 4.

As can be seen from Table 4, the GOHBA has a rank count of 20 and an average rank of 2.0, which indicates that the GOHBA has the best overall performance among the 10 test functions mentioned above. The results of Friedman’s test once again prove that the GOHBA outperforms other algorithms.

### 3.6. Wilcoxon Symbolic Rank Calibration

The Wilcoxon signed-rank test is a nonparametric hypothesis test that determines the difference in performance between two algorithms by evaluating whether the results of the two algorithms on the benchmark function are different [33]. In this section, we compare the GOHBA with seven other algorithms using the Wilcoxon signed-rank test to calculate the *p*-value and significance sign (h) of the Wilcoxon signed-rank test. The significance level was set at 0.05, and when p<0.05, = 1, there is a significant difference between the performance of the GOHBA and the compared algorithms. When p>0.05, = 0, there is no significant difference in performance between the GOHBA and the comparison algorithm. In addition, when *p* is NaN, we believe that the algorithm can achieve the same results as the GOHBA. The results of the Wilcoxon signed-rank test between the GOHBA and the other seven algorithms on 24 test functions are shown in Table 5.

As shown in Table 5, the GOHBA significantly outperforms the other algorithms on the single-peak test functions $F_{1}$~$F_{7}$ with a *p*-value less than 0.05 and an h-value equal to 1. On the multimodal test functions such as $F_{12}$,$F_{13}$, etc., there is also a significant difference with the other functions. This means that on most of the test functions, the GOHBA is significantly different from the other 7 algorithms. Therefore, we conclude that the GOHBA significantly outperforms the 7 algorithms with which it is compared.

### 3.7. Convergence Analysis

**Figure 5:**
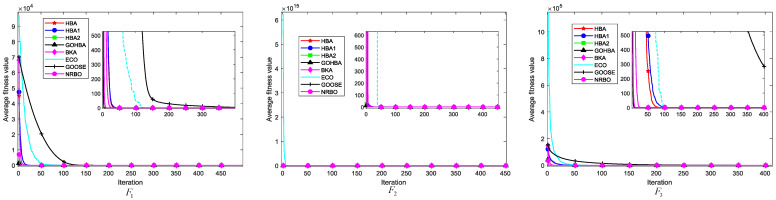

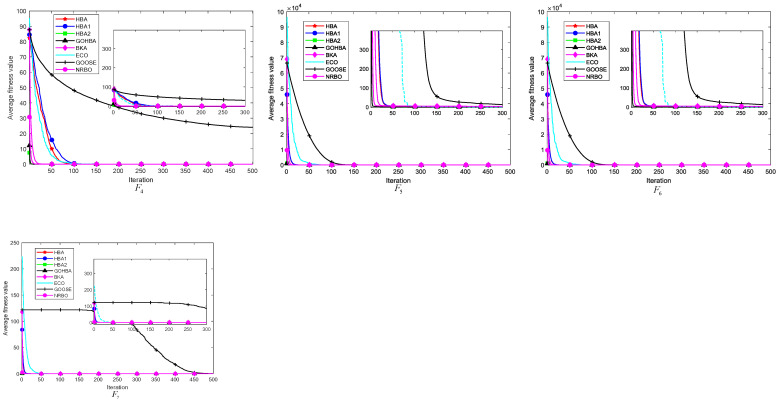
Average convergence curves of GOHBA with its comparison algorithm on single-peak test functions.

The convergence analysis of the algorithms assesses the nature of the algorithms based on their ability to progressively approach the goal or steady state during the iteration process. p/t was set to 30/500 and 50 independent experiments were performed. Figure 4 shows the average convergence curves of the GOHBA versus the comparison algorithms for 50 cycles on seven single-peak test functions. Figure 5 shows the average convergence curve of the GOHBA with the comparison algorithm cycled 50 times on 6 multimodal test functions.

Figure 5 shows the average convergence curves of the GOHBA and its comparison algorithm on $F_{1}$ to $F_{7}$. As can be seen from Figure 4, the algorithm converges to a smaller value on all test functions. The blue line in the figure is ECO, which is located in the upper right of the graphs of $F_{2}$,$F_{3}$ and $F_{5}$, indicating slow convergence and poor convergence performance. The black line is GOOSE, which lies above all the curves on $F_{1}$,$F_{4}$ and $F_{6}$, indicating that it does not converge as fast as the other algorithms. On $F_{7}$, the blue and black lines alternate above in different regions. Among the curves above $F_{1}$~$F_{7}$, the curves of the GOHBA are all located at the bottom of the chart, indicating that the average convergence curve of the GOHBA is lower than that of other algorithms, suggesting that the GOHBA has a faster convergence rate.

**Figure 6:**
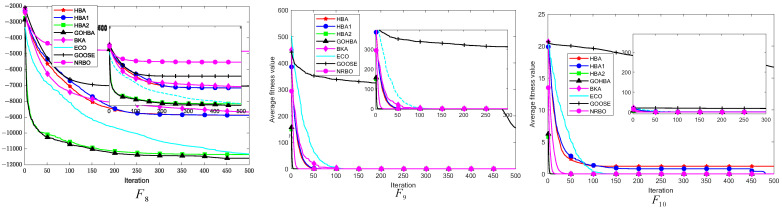

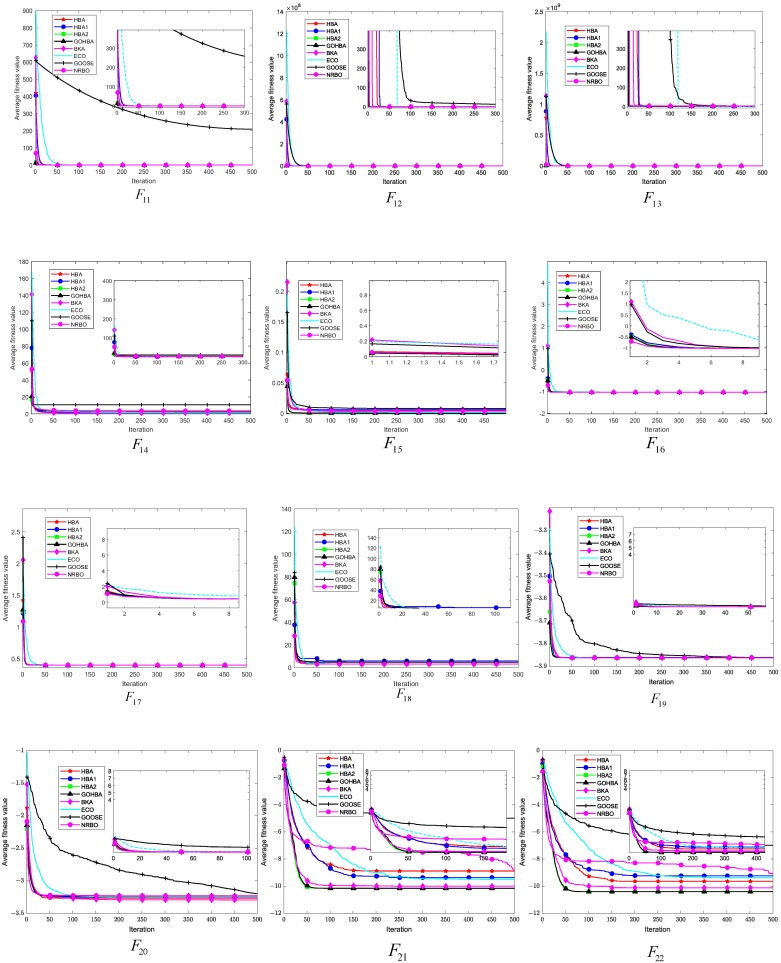

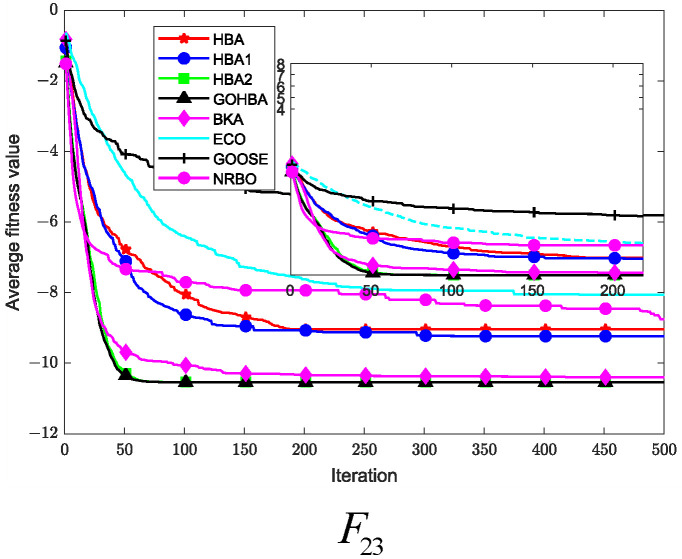
Average convergence curves of GOHBA with its comparison algorithms on multimodal test functions.

Figure 6 shows the average convergence curves of the GOHBA and its comparison algorithms on $F_{8}$~$F_{23}$. The black line in the figure is GOOSE, which lies on top of all curves from $F_{8}$ to $F_{13}$ and has slow convergence and poor convergence performance. The light blue line is ECO, which is on the curves except $F_{13}$,$F_{16}$,$F_{17}$, and $F_{21}$ to $F_{23}$, and has slow convergence. The average convergence curve of the GOHBA is lower than the other functions on all functions. Overall, the GOHBA has higher convergence efficiency compared to other algorithms, indicating that the improvements to the HBA are effective and enhance the convergence performance of the HBA.

### 3.8. Stability Analysis

**Figure 7:**
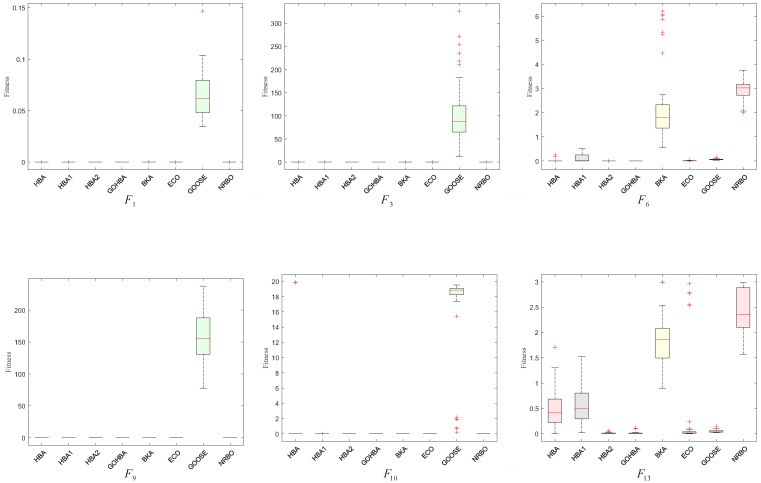

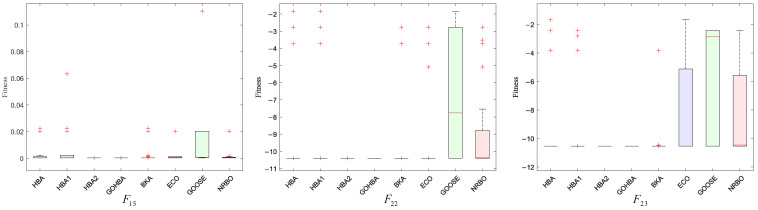
The boxplots of GOHBA and the comparison algorithms.

In this section, box-and-line diagrams are used to analyze the stability of each algorithm, and each algorithm is run independently 50 times. The first 6 single-peak test functions and the first 6 multi-peak test functions are selected for comparison. The box-and-line diagram and comparison algorithms for the GOHBA are shown in Figure 7.

Figure 7 shows a boxplot of the GOHBA with the other 7 meta-heuristics run independently for 50 experiments. The GOHBA shows the best stability on $F_{1}$,$F_{3}$,$F_{6}$,$F_{9}$,$F_{10}$,$F_{13}$,$F_{15}$,$F_{22}$ and $F_{23}$. On $F_{1}$,$F_{3}$,$F_{9}$,$F_{10}$,$F_{15}$,$F_{23}$, the GOHBA is stable with individual outliers. And the improvement of the GOHBA is not obvious on $F_{13}$. The GOHBA shows better stability in the experiment.

Based on the above analysis, the GOHBA has the most stable performance. Tent chaotic population initialization enhanced the population diversity and initialized the population quality of the GOHBA. Designing different density factors enhanced the population diversity. The Golden Sine Strategy enhanced the GOHBA’s ability to search the problem space, effectively increasing its ability to jump out of the local optimum. These strategies enabled the GOHBA to converge to the global optimum solution stably over multiple runs.

## 4. GOHBA Application

In this section, two constrained optimization problems derived from practical engineering scenarios have been chosen to evaluate the performance of the proposed GOHBA. Furthermore, to underscore the applicability of the GOHBA in real-world contexts, it will be utilized in the domain of path planning.

### 4.1. Application to Engineering Design Problems

The two engineering optimization problems discussed in this section are the robot gripper problem and the speed reducer design problem. These optimization issues encountered in the real world are typically characterized as constrained optimization problems, necessitating the employment of meta-heuristic approaches equipped with techniques for handling constraints. Owing to its simplicity and ease of implementation, the penalty function method is one of the widely adopted methods for handling constraints [34]. The basic idea of the penalty function method is to construct a certain penalty function according to the characteristics of the constraints and add it to the objective function to establish the unconstrained problem where we apply the penalty function method to handle the constraints. The performance of the GOHBA is evaluated by comparing it with other evolutionary algorithms. The number of tests is 30, the population size is 30, and the maximum number of iterations is 500.

In this study, to effectively deal with constrained optimization problems, we employed a static selection method to determine the penalty coefficient, setting it at $10^{100}$. This value was chosen by taking into account the problem scale, the strictness of constraints, and the results of preliminary experiments. The aim was to balance the objective function and constraints during the optimization process, ensuring that the algorithm searches efficiently while strictly satisfying the constraints.

#### 4.1.1. Robot Gripper Design Problem

**Figure 8:**
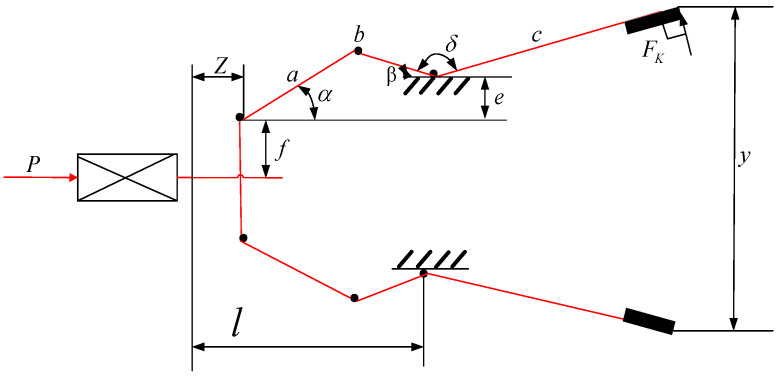
Robot gripper design optimization problem diagram.

The robot gripper design problem is shown in Figure 8 [35] and the goal is to minimize the difference between the maximum and minimum forces applied to the gripper by the range of gripper end displacements. The problem consists of seven design variables relevant to the robot: including the gripper width (*a*); the gripper width (*b*); the gripper thickness (*c*); the maximum opening angle (δ); the vertical offset(*e*); the drive parameter (*f*); the length of the gripper (*l*). The mathematical model is shown in Equation (19):

Consider variable

$$x=(x_{1},x_{2},x_{3},x_{4},x_{5},x_{6},x_{7})=(a,b,c,e,f,l,\delta )$$

Minimize $f(x)=-\underset{z}{\min}\;F_{K}(x,z)+\underset{z}{\max}\;F_{K}(x,z)$

Subject to

(19) $$g_{1}(x)=-Y_{\min}+y(x,Z_{\max})\leq 0,\;g_{2}(x)=-y(x,Z_{\max})\leq 0,\;g_{3}(x)=Y_{\max}-y(x,0)\leq 0,\;g_{4}(x)=y(x,0)-Y_{G}\leq 0,\;g_{5}(x)=l^{2}+e^{2}-{\left(a+b\right)}^{2}\leq 0,\;g_{6}(x)=b^{2}-{\left(a-e\right)}^{2}-{\left(l-Z_{\max}\right)}^{2}\leq 0,\;g_{7}(x)=Z_{\max}-l\leq 0,$$

where $\alpha =\cos^{-1}(\frac{a^{2}+g^{2}-b^{2}}{2ag})+\phi ,g=\sqrt{e^{2}+{\left(z-l\right)}^{2}}$,$\beta =\cos^{-1}(\frac{b^{2}+g^{2}-a^{2}}{2ag})-\phi$,$\phi =\tan^{-1}(\frac{e}{l-z})$,y(x,z)=2(f+e+csin(β+δ)),$F_{K}=\frac{Pb\sin(\alpha +\beta )}{2c\cos(\alpha )}$,$Y_{\min}=50$,$Y_{\max}=100$,$Y_{G}=150$,$Z_{\max}=100$,P=100.

With bounds

0≤e≤50,100≤c≤200,10≤f,a,b≤150,1≤δ≤3.14,100≤l≤300.

The GOHBA is used to optimize the robot gripper problem and is compared with seven other algorithms. The experimental results are shown in Table 6, which indicates that the GOHBA gives better values and better performance on this problem.

#### 4.1.2. Speed Reducer Design Problem

**Figure 9:**
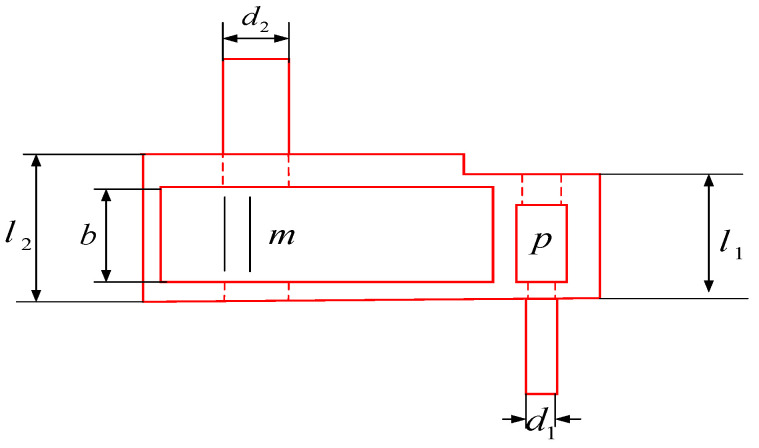
Speed reducer design problem diagram.

The speed reducer design problem is an engineering design problem [36]. It has seven design variables, as shown in Figure 9. The main objective of this design problem is to minimize the weight of the reducer while satisfying the following constraints: bending stresses on the gear teeth, surface pressure, lateral deflection of the shaft, and stresses on the shaft. There are seven design variables ($z_{1}$-$z_{7}$) such as face width (b), module of the teeth (m), number of gear teeth (p), length of the first shaft between the bearings ($l_{1}$), length of the second shaft between the bearings ($l_{2}$), diameter of the first shaft ($d_{1}$), and diameter of the second shaft ($d_{2}$). The mathematical formulation of the problem is described as shown in Equation (20):

$$C\mathrm{onsider}\bar{z}=[z_{1},z_{2},z_{3},z_{4},z_{5},z_{6},z_{7}]=[b,m,p,l_{1},l_{2},d_{1},d_{2}]\;\begin{array}{l} Minimizef(\bar{z})=0.7854z_{1}z_{2}^{2}(3.3333z_{3}^{2}+14.9334z_{3}-43.0934)-1.508z_{1}(z_{6}^{2}+z_{7}^{2})\\ +7.4777(z_{6}^{3}+z_{7}^{3})+0.7854(z_{4}z_{6}^{2}+z_{5}z_{7}^{2}), \end{array}$$

Subject to:

(20) $$g_{1}(\bar{z})=\frac{27}{z_{1}z_{2}^{2}z_{3}^{2}}-1\leq 0,\;g_{2}(\bar{z})=\frac{397.5}{z_{1}z_{2}^{2}z_{3}^{2}}-1\leq 0,\;g_{3}(\bar{z})=\frac{1.93z_{4}^{3}}{z_{2}z_{7}^{4}z_{3}}-1\leq 0,\;g_{4}(\bar{z})=\frac{1.93z_{4}^{3}}{z_{2}z_{7}^{4}z_{3}}-1\leq 0,\;g_{5}(\bar{z})=\frac{{\left[{\left(745({z_{4}/z_{2}z}_{3})\right)}^{2}+16.9\times 10^{6}\right]}^{1/2}}{110z_{6}^{3}}-1\leq 0,\;g_{6}(\bar{z})=\frac{{\left[{\left(745(z_{5}/z_{2}z_{3})\right)}^{2}+157.5\times 10^{6}\right]}^{1/2}}{85z_{7}^{3}}-1\leq 0,\;g_{7}(\bar{z})=\frac{z_{2}z_{3}}{40}-1\leq 0,\;g_{8}(\bar{z})=\frac{5z_{2}}{z_{1}}-1\leq 0,\;g_{9}(\bar{z})=\frac{z_{1}}{12z_{2}}-1\leq 0,\;g_{10}(\bar{z})=\frac{1.5z_{6}+1.9}{z_{4}}-1\leq 0,\;g_{11}(\bar{z})=\frac{1.1z_{7}+1.9}{z_{5}}-1\leq 0,$$

where,

$$2.6\leq z_{1}\leq 3.6,0.7\leq z_{2}\leq 0.8,17\leq z_{3}\leq 28,7.3\leq z_{4}\leq 8.3,7.3\leq z_{5}\leq 8.3,2.9\leq z_{6}\leq 3.9,5.0\leq z_{7}\leq 5.5$$

The GOHBA was used to optimize the speed reducer design problem and was compared with seven other algorithms. The experimental results are shown in Table 7. The GOHBA reached the optimization. It shows that the GOHBA has the best light weighting effect for the reducer with the constraints satisfied.

### 4.2. Robot Path Planning with GOHBA

Path planning is a vital component in the domain of autonomous mobile robot navigation and planning. Grid-based approaches are frequently employed for modeling the robot’s surroundings due to their simplicity and straightforward implementation, as referenced in [37,38]. This section showcases the utilization of the GOHBA (global optimization HBA) with 2D raster maps to highlight its practical use.

#### Simulation of Robot Path Planning

In this section, the GOHBA will be applied to the plotted raster maps: MAP1, MAP2, and MAP3. The results, convergence curves, and paths given in this section are the only ones available for this study.

The size of MAP1 is 20 × 20, the size of MAP2 is 30 × 30, and the size of MAP3 is 40 × 40. All three maps start at the bottom left corner and end at the top right corner of the map. In this study, the population size N and the maximum number of iterations T are set to 30 and 50, respectively. Based on this setting, a visual comparison of the algorithm performance obtained by the eight algorithms on the three maps as well as a comparison of the convergence curves are shown in Figure 9. From the convergence curves in Figure 9, it can be seen that the GOHBA has a faster convergence rate than the other algorithms in solving the robot path planning problem.

Table 8 gives the comparison statistics of GOHBA with the other seven algorithms (BKA, ECO, GOOSE, HBA, HBA1, HBA2, NRBO) on three maps. From Table 8, we know that all algorithms converge to the global optimum on MAP1, MAP2, and MAP3. On the mean metric of MAP1, the GOHBA reaches the optimality and the other algorithms are worse than the GOHBA on average. On the mean metrics of MAP2 and MAP3, the GOHBA’s results also reach optimality. For the standard deviation of MAP1, the GOHBA results do not reach optimality. However, for the standard deviation of MAP2 and MAP3, the GOHBA results are also optimized. In summary, the results obtained by the GOHBA are more in line with the requirements of the map.

**Figure 10:**
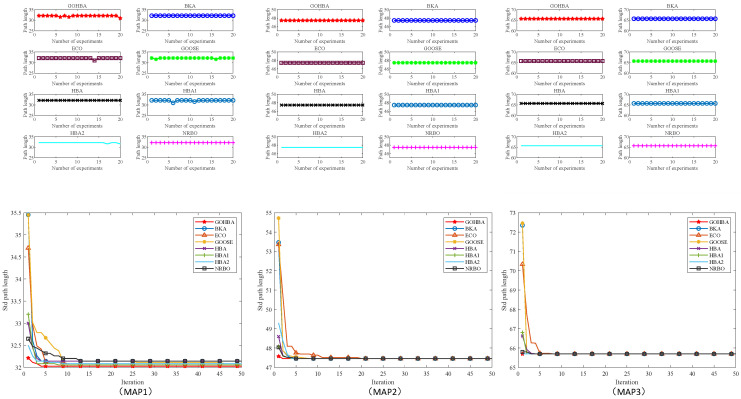
Visual comparison of algorithm performance and convergence curves for 8 algorithms.

Figure 10 gives the comparison statistics of the GOHBA with the other seven algorithms on maps MAP1, MAP2, and MAP3. From the figure, it can be seen that the GOHBA can reach the optimal path the fastest in the solution process of MAP1, MAP2, and MAP3. It shows that the GOHBA can approach the optimal solution stably and stabilize quickly, while other algorithms encounter difficulties in finding the optimal solution. In the Mean index, the results of the GOHBA on all three maps are better than other algorithms and reach the optimum. On the Std metric, the standard deviation of the GOHBA is not optimal during the solution of MAP1. On the other hand, the standard deviation of the GOHBA reaches the optimization in two maps, MAP2 and MAP3. To summarize, the GOHBA is more in line with the requirements of path planning and has the best results.

**Figure 11:**
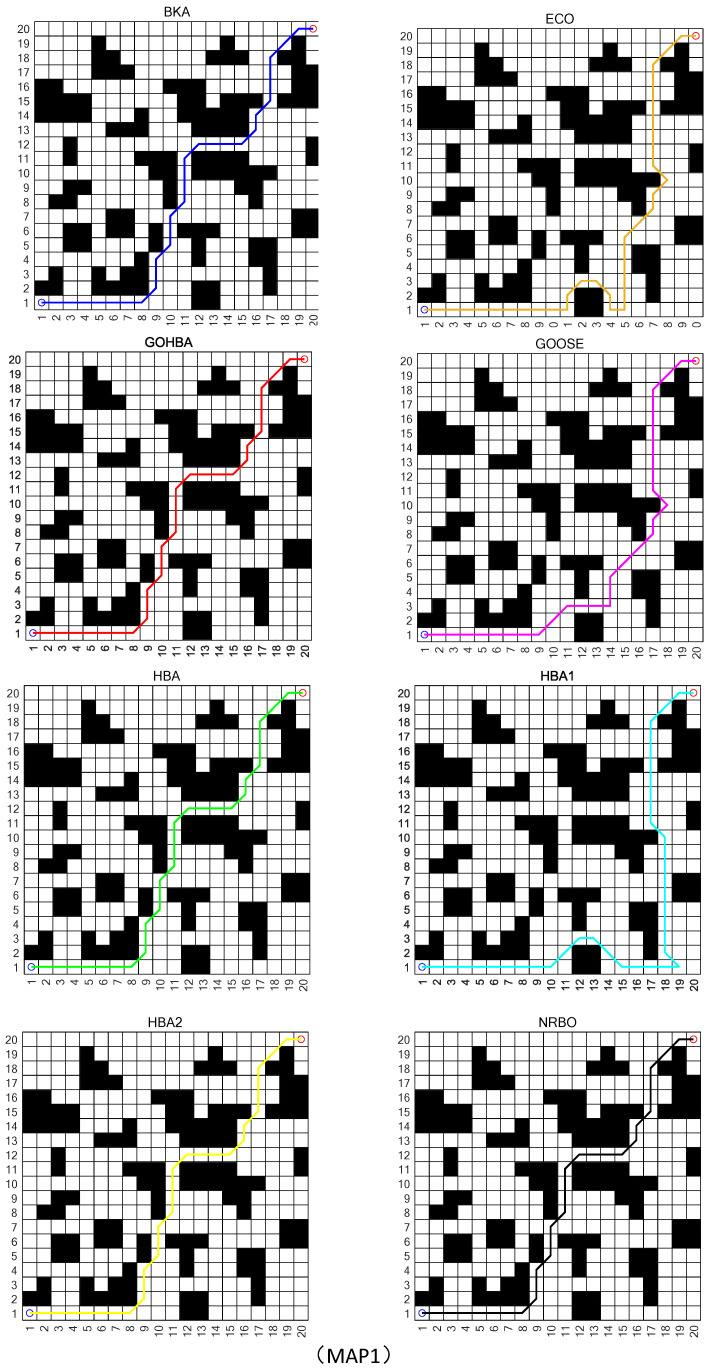

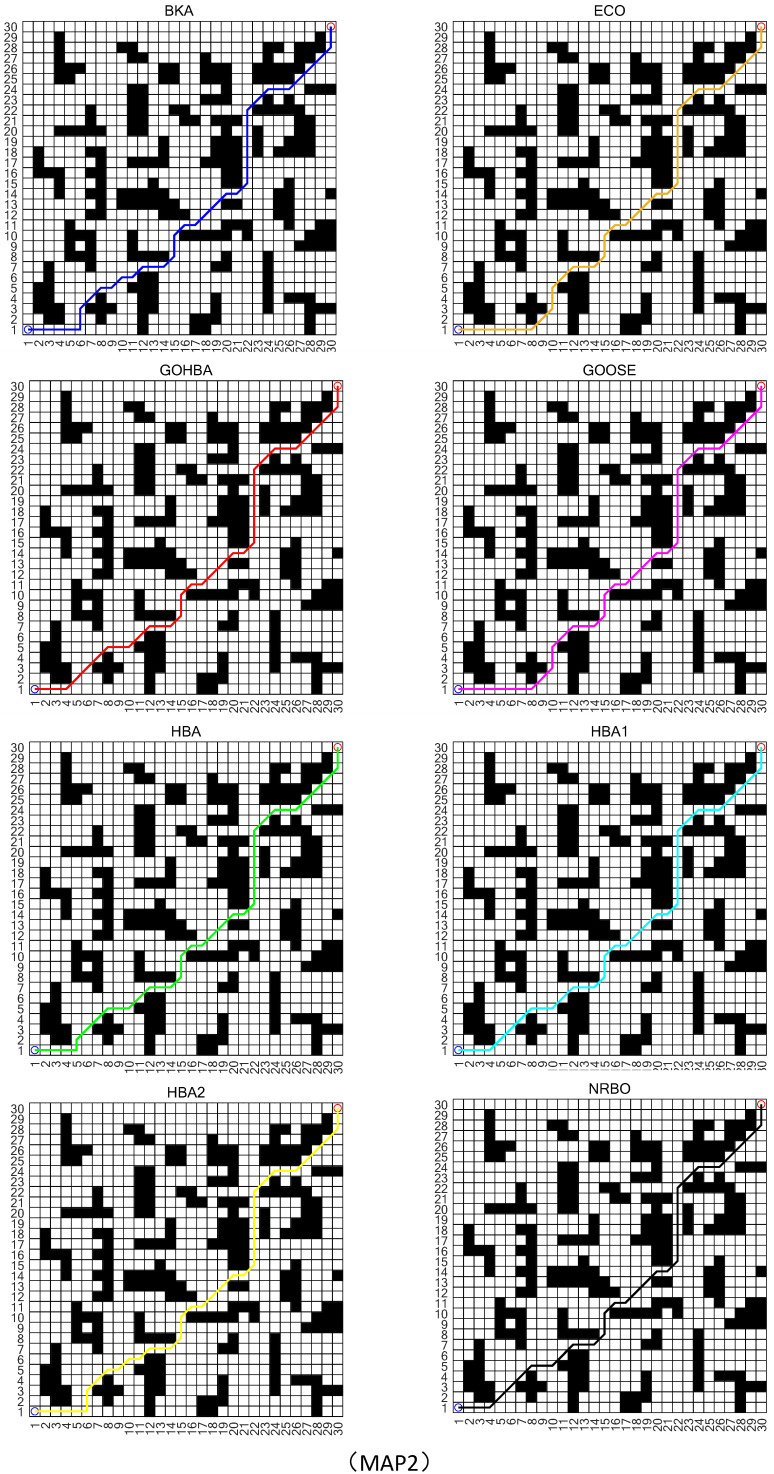

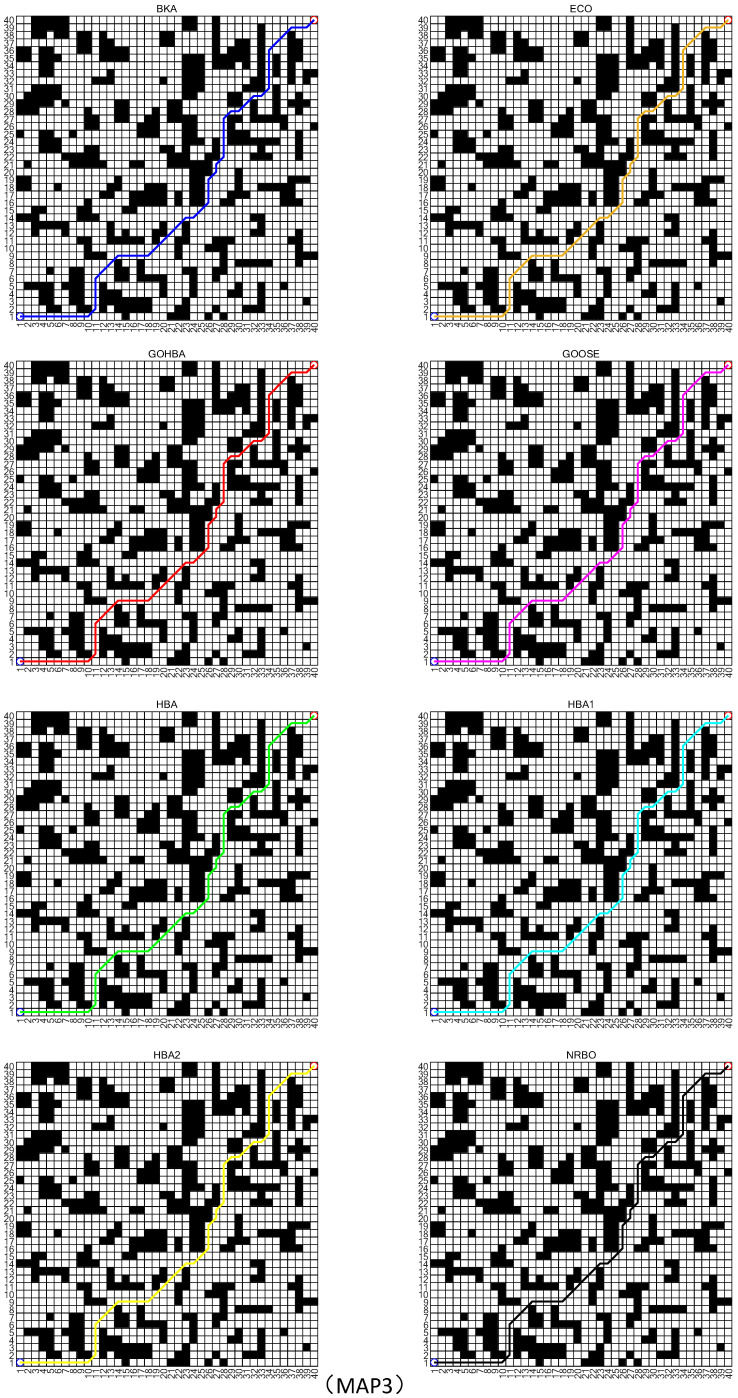
The optimal path on 3 maps with 8 algorithms.

Figure 11 shows the optimal paths found by the GOHBA algorithm and the other seven algorithms on the three maps MAP1, MAP2, and MAP3 from the starting point of the blue circle in the lower left corner to the end point of the red circle in the upper right corner; it can be noticed from the figure that the different colors correspond to the algorithm names in the upper left corner of the picture and show the corresponding path trajectories on the maps. Comparison experiments show that GOHBA has good convergence in solving the robot path planning problem.

### 4.3. GOHBA Discussion

We apply the GOHBA to two real-world engineering optimization problems and quadruped robot path planning. The robot gripper problem is a complex issue in mechanical structural engineering. It involves seven design variables, represented as six nonlinear design constraints related to the robot, making it challenging to solve. The GOHBA has demonstrated optimization values that better align with the requirements of the robot gripper problem. The gear reducer design problem is an engineering design issue. It involves seven design variables and aims to reduce the weight of the gear reducer while satisfying the following constraints. GOHBA achieves an optimal solution for quadruped robot path planning. We conducted experiments on three different maps. The mean and standard deviation can better help us evaluate the performance of the GOHBA and the other seven algorithms in path planning on different maps. In terms of standard deviation, the GOHBA is more stable on the map. From the experimental results, it can be seen that the GOHBA has higher stability and convergence speed in quadruped robot path planning.

In summary, the GOHBA has obvious advantages in practical engineering problems as well as path planning problems. Tent chaotic mapping initialization enhances the population diversity and initialized population quality of the HBA. Secondly, the introduction of a new density factor enhances the algorithm to explore more extensively in the whole solution space, especially in the early stage of the algorithm, which can effectively avoid premature convergence to local optimal solutions. Finally, the introduction of the Golden Sine Strategy improves the global search capability of the HBA, accelerates the convergence speed, and helps to avoid falling into local optima. However, despite its excellent performance, the GOHBA still has some shortcomings, such as failing to achieve the expected efficiency and accuracy on some path planning maps, as well as failing to achieve the best results on some engineering optimization projects.

## 5. Conclusions

In this study, we propose a global optimization honey badger algorithm GOHBA (global optimization HBA) to solve the challenges of the honey badger algorithm, which is prone to fall into local optimums, slow convergence, and insufficient global search capability, and further improve the performance of the honey badger algorithm.

We have made three improvements to the honey badger algorithm. First, we introduced Tent chaotic mapping initialization to enhance the population diversity and initialized population quality of the HBA. The reason for choosing the Tent chaotic algorithm is that it can generate Gaussian-distributed random numbers with better randomness and some exploration. Secondly, the introduction of the new density factor enhances the algorithm to explore more extensively in the whole solution space, especially in the early stage of the algorithm, which can effectively avoid premature convergence to local optimal solutions. Finally, the introduction of the Golden Sine Strategy improves the global search capability of the HBA, accelerates the convergence speed, and helps to avoid falling into local optima.

We also conducted experiments on the GOHBA on 23 test functions and compared its performance with its HBA variant and four meta-heuristic algorithms, counting the mean and standard deviation of the algorithm’s optimized search. The experimental results show that the GOHBA is optimal in terms of the mean in 14 test functions, optimal in terms of standard deviation in 11 test functions, and ranks second in terms of mean and standard deviation in seven test functions. The experimental results show that the algorithm has high stability and accuracy, and can jump out of the local optimum faster.

The optimization capability of the global optimization honey badger algorithm is verified by applying the GOHBA to two real engineering design problems. On the robot gripper problem, the GOHBA optimized values are more in line with the requirements of the robot gripper problem, which proves that the GOHBA has a high convergence rate; on the reducer problem, the GOHBA has the best light weighting effect for the reducer with the constraints satisfied. Compared with the other seven algorithms, the GOHBA (global optimization HBA) has higher accuracy and a faster convergence rate on the path planning problem. The results show that the average value obtained by the GOHBA on all three maps is optimal. The standard deviation obtained by the GOHBA on top of MAP2 and MAP3 is optimal. It can be seen that the GOHBA improves the searchability of the population, and has a better ability to jump out of the local optimum, faster convergence, and better stability. The above experiments further illustrate the effectiveness of the GOHBA. The results show that the GOHBA is optimal on two real engineering design problems. On the path planning problem, the GOHBA improves the search ability of the population, and has better ability to jump out of the local optimum, faster convergence, and better stability.

Although the GOHBA performs well in optimization problems, its main limitation lies in the fact that the introduced GSA mechanism improves the computational complexity but also increases the time consumption. Looking forward to future research directions, it is expected that this problem can be effectively mitigated by implementing a parallelization procedure, which can significantly reduce the computational time consumption while maintaining the optimization performance of the algorithm, and further improve the feasibility and efficiency of the GOHBA in practical applications.

Future research on the global optimization honey badger algorithm (GOHBA) can focus on expanding its applications and enhancing performance. By combining the GOHBA with machine learning algorithms, such as optimizing SVM hyperparameters or neural network structures, we can improve model accuracy in tasks like image recognition. Extending the GOHBA to multi-objective optimization and dynamic environments will broaden its applicability to complex problems. Leveraging parallel computing technologies, such as GPUs and Spark, can boost computational efficiency for large-scale problems. Additionally, applying the GOHBA to fields like industrial manufacturing, energy management, logistics, and finance can address practical challenges and enhance system performance.

To further enhance the adaptability and performance of the GOHBA across diverse problem sets, future research should focus on automated parameter tuning. Currently, parameter settings often require manual adjustment based on empirical knowledge and trial-and-error, which can be time-consuming and suboptimal. Developing automated methods for parameter tuning is essential. Techniques such as Bayesian optimization or machine learning-based approaches could dynamically adjust parameters during the optimization process, ensuring that the GOHBA adapts optimally to different problem characteristics and complexities. This would increase the GOHBA’s robustness and applicability, from engineering designs to financial predictions. Additionally, integrating the GOHBA with other advanced optimization methods could further boost its performance in solving complex real-world problems.

## Figures and Tables

**Table 1:** Test functions.

Function	D	R	*f* (x*)
$f_{1}\left(x\right)=\sum_{i=1}^{n}x_{i}^{2}$	30	[−100,100]	0
$f_{2}\left(x\right)=\sum_{i=1}^{n}\left|x_{i}\right|+\prod_{i=1}^{n}\left|x_{i}\right|$	30	[−10,10]	0
$f_{3}\left(x\right)=\sum_{i=0}^{n-1}{\left\{\sum_{j=0}^{j<i}x_{i}\right\}}^{2}$	30	[−100,100]	0
$f_{4}\left(x\right)=\max_{i}\left\{\left|x_{i}\right|,1\leq i\leq n\right\}$	30	[−100,100]	0
$f_{5}\left(x\right)=\sum_{i=1}^{D}\left|x_{i}\right|$	30	[−30,30]	0
$f_{6}\left(x\right)=\sum_{i=1}^{n}{\left(\left[x_{i}+0.5\right]\right)}^{2}$	30	[−100,100]	0
$f_{7}\left(x\right)=\sum_{i=1}^{n}ix_{i}^{4}+random[0,1)$	30	[−1.28,1.28]	0
$f_{8}\left(x\right)=\sum_{i=1}^{n}-xi\sin\left(\sqrt{\left|x_{i}\right|}\right)$	30	[−500,500]	−12569.4
$f_{9}\left(x\right)=\sum_{i=1}^{n}\left[x_{i}^{2}-10\cos\left(2\pi x_{i}\right)+10\right]$	30	[−5.12,5.12]	0
$f_{10}\left(x\right)=-20exp\left(-0.2\sqrt{\left(1/n\right)\times \sum_{i=1}^{n}x_{i}^{2}}\right)-exp(\left(1/n\right)\times \sum_{i=1}^{n}\cos\left(2\pi x_{i}\right))+20+e$	30	[−32,32]	0
$f_{11}=\left(1/400\right)\times \sum_{i=1}^{n}x_{i}^{2}-\prod_{i=1}^{n}\cos\left(x_{i}/\sqrt{x+1}\right)+1$	30	[−600,600]	0
$f_{12}\left(x\right)=\left(\pi /n\right)\times \left\{10\sin\left(\pi y_{1}\right)+{\left(y_{n}-1\right)}^{2}+\sum_{i=1}^{n-1}{\left(y_{i}-1\right)}^{2}[1+10\sin^{2}\left(\pi y_{i+1}\right)]\right\}$ $y_{i}=1+\left(x_{i}+1\right)/4$ $u(x_{i},a,k,m)=\left\{\begin{array}{c} k{\left(x_{i}-a\right)}^{m}\;\;\;\;x_{i}>a\\ 0\;\;\;-a<x_{i}<1\\ k{\left({-x}_{i}-a\right)}^{m}\;\;\;\;x_{i}<-a \end{array}\right.$	30	[−50,50]	0
$f_{13}\left(x\right)=0.1\left\{\sum_{i=1}^{n}{\left(x_{i}-1\right)}^{2}\left[1+\sin^{2}\left(3\pi x_{1}+1\right)\right]\sin^{2}\left(3\pi x_{1}\right)+{\left(x_{n}-1\right)}^{2}[1+\sin^{2}\left(2\pi x_{ni}\right)]\right\}+\sum_{i=1}^{n}u\left(x_{i},5,100,4\right)$	30	[−50,50]	0
$f_{14}\left(x\right)={\left(1/500+\sum_{j=1}^{25}\left(1/\left(j+\sum_{i=1}^{2}{\left(x_{i}-a_{ij}\right)}^{6}\right)\right)\right)}^{-1}$	2	[−65,65]	1
$f_{15}\left(x\right)=\sum_{i=1}^{11}{\left[a_{i}-x_{1}\left(b_{i}^{2}+b_{i}x_{2}\right)/\left(b_{i}^{2}+b_{i}x_{3}+x_{4}\right)\right]}^{2}$	4	[−5,5]	0.00003075
$f_{16}\left(x\right)=4x_{1}^{2}-2.1x_{1}^{4}+x_{1}^{6}/3+x_{1}x_{2}-4x_{2}^{2}+4x_{2}^{4}$	2	[−5,5]	−1.0316285
$f_{17}\left(x\right)={\left(x^{2}-\left(5.1/4\pi^{2}\right)x_{1}^{2}+\left(5/\pi \right)x_{1}-6\right)}^{2}+10\left(1-\left(1/8\pi \right)\right)cosx_{1}+10$	2	[−5,5]	0.398
$f_{18}\left(x\right)=\left[1+{\left(x_{1}+x_{2}+1\right)}^{2}\times \left(19-14x_{1}+3x_{1}^{2}-14x_{2}+6x_{1}x_{2}+3x_{2}^{2}\right)\right]\cdot \left[30+{\left(2x_{1}-3x_{2}\right)}^{2}\times \left(18-32x_{1}+12x_{1}^{2}+48x_{2}-36x_{1}x_{2}+27x_{2}^{2}\right)\right]$	2	[−2,2]	3
$f_{19}\left(x\right)=-\sum_{i=1}^{4}c_{i}\exp(-\sum_{j=1}^{3}a_{ij}{\left(x_{j}-p_{ij}\right)}^{2})$	3	[0,1]	−3.86
$f_{20}\left(x\right)=-\sum_{i=1}^{4}c_{i}\exp(-\sum_{j=1}^{6}a_{ij}{\left(x_{j}-p_{ij}\right)}^{2})$	6	[0,1]	−3.32
$f_{21}\left(x\right)=-\sum_{i=1}^{5}{\left[\left(x-a_{i}\right){\left(x-a_{i}\right)}^{T}+c_{i}\right]}^{-1}$	4	[0,10]	−10
$f_{22}\left(x\right)=-\sum_{i=1}^{7}{\left[\left(x-a_{i}\right){\left(x-a_{i}\right)}^{T}+c_{i}\right]}^{-1}$	4	[0.10]	−10
$f_{23}\left(x\right)=-\sum_{i=1}^{10}{\left[\left(x-a_{i}\right){\left(x-a_{i}\right)}^{T}+c_{i}\right]}^{-1}$	4	[0,10]	−10

**Table 2:** p, t sensitivity analysis.

Function	Criterion	*p/t* 60/250	*p/t* 30/500	*p/t* 15/1000
*F*_1_	Mean Std Rank	1.7142 × 10^−283^ 0 2	0 0 2	0 0 2
*F*_2_	Mean Std Rank	1.5485 × 10^−145^ 8.7921 × 10^−145^ 3	1.2544 × 10^−273^ 0 1.5	0 0 1.5
*F*_3_	Mean Std Rank	2.5563E−273 0 2	0 0 2	0 0 2
*F*_4_	Mean Std Rank	2.2952 × 10^−143^ 9.0280 × 10^−143^ 3	1.0078 × 10^−268^ 0 1.5	0 0 1.5
*F*_5_	Mean Std Rank	2.5151 × 10^1^ 4.6295 × 10^−1^ 2	2.4791 × 10^1^ 5.2181 × 10^-1^ 3	2.4916 × 10^1^ 3.6635 × 10^−1^ 1
*F*_6_	Mean Std Rank	1.0971 × 10^−3^ 6.6149 × 10^−4^ 3	2.3806 × 10^−04^ 1.7511 × 10^−04^ 2	9.5836 × 10^−5^ 7.7331 × 10^−5^ 1
*F*_7_	Mean	1.5468 × 10^−4^	1.3085 × 10^−4^	1.6314 × 10^−4^
	Std	1.3565 × 10^−4^	9.6256 × 10^−5^	1.5177 × 10^−4^
	Rank	2	1	3
*F*_8_	Mean Std Rank	−1.1565 × 10^4^ 8.7136 × 10^2^ 3	−1.1419 × 10^4^ 7.5399 × 10^2^ 1	−1.1425 × 10^4^ 8.7099 × 10^2^ 2
*F*_9_	Mean Std Rank	0 0 2	0 0 2	0 0 2
*F*_10_	Mean Std Rank	4.4409 × 10^−16^ 0 2	4.4409 × 10^−16^ 0 2	4.4409 × 10^−16^ 0 2
*F*_11_	Mean Std Rank	0 0 2	0 0 2	0 0 2
*F*_12_	Mean Std Rank	1.3269 × 10^−4^ 7.5080 × 10^−5^ 3	3.1881E × 10^−5^ 2.6201 × 10^−5^ 1	1.9846 × 10^−5^ 3.1180 × 10^−5^ 2
*F*_13_	Mean Std Rank	5.7978 × 10^−3^ 6.7686 × 10^−3^ 1	9.7015 × 10^−3^ 2.0111 × 10^−2^ 2	1.5350 × 10^−2^ 2.1065 × 10^−2^ 3
*F*_14_	Mean Std Rank	2.3088 3.1913 1	2.9375 3.5261 2	4.0693 4.2901 3
*F*_15_	Mean Std Rank	3.0756 × 10^−4^ 1.3235 × 10^−7^ 1	3.0780 × 10^−4^ 8.7970 × 10^−7^ 2	4.6868 × 10^−4^ 1.1350 × 10^−3^ 3
*F*_16_	Mean Std Rank	−1.0316 3.7532 × 10^−16^ 3	−1.0316 3.5037 × 10^−16^ 2	−1.0316 3.4164 × 10^−16^ 1
*F*_17_	Mean Std Rank	3.9789 × 10^−1^ 0 2	3.9789 × 10^−1^ 0 2	3.9789 × 10^−1^ 0 2
*F*_18_	Mean Std Rank	3.0000 2.6773 × 10^−15^ 1	3.5400 3.8184 2	6.7800 13.373 3
*F*_19_	Mean Std Rank	−3.8622 2.1599 × 10^−3^ 2	−3.8626 1.1146 × 10^−03^ 1	−3.8620 2.3885 × 10^−03^ 3
*F*_20_	Mean Std Rank	−3.2282 6.3648 × 10^−02^ 1	−3.2142 8.4419 × 10^−2^ 2	−3.2353 8.9149 × 10^−2^ 3
*F*_21_	Mean Std Rank	−10.153 2.3119 × 10^−15^ 3	−10.153 1.1061 × 10^−15^ 1	−10.153 1.6833 × 10^−15^ 2
*F*_22_	Mean Std Rank	−10.403 1.4355 × 10^−15^ 1	−10.403 2.1682 × 10^−15^ 2	−10.250 1.0800 3
*F*_23_	Mean	−10.536	−10.536	−10.536
	Std	2.2697 × 10^−15^	2.2555 × 10^−15^	2.4864 × 10^−15^
	Rank	2	1	3
Rank-Count Ave-Rank Overall-Rank		47.00 2.04 2	40.00 1.74 1	51.00 2.22 3

**Table 3:** Experimental results of the GOHBA and its comparative algorithms.

Function	Algorithm	Mean	Std	Function	Algorithm	Mean	Std
*F*_1_	HBA HBA1 HBA2 GOHBA BKA ECO GOOSE NRBO	9.5334 × 10^−136^ 1.8327 × 10^−147^ 0 0 1.7665 × 10^−73^ 4.7616 × 10^−43^ 6.6684 × 10^−2^ 4.8704 × 10^−280^	3.1770 × 10^−135^ 8.6713 × 10^−147^ 0 0 1.2490 × 10^−72^ 3.2749 × 10^−42^ 2.2264 × 10^−02^ 0	*F*_13_	HBA HBA1 HBA2 GOHBA BKA ECO GOOSE NRBO	0.50283 0.54545 6.3470 × 10^−3^ 1.0836 × 10^−2^ 1.8507 0.29398 4.9868 × 10^−2^ 2.4079	0.35069 0.33952 1.0825 × 10^-2^ 2.3800 × 10^-2^ 0.48175 0.81989 2.7722 × 10^-2^ 0.42596
*F*_2_	HBA HBA1 HBA2 GOHBA BKA ECO GOOSE NRBO	1.1649 × 10^−72^ 3.1952 × 10^−77^ 5.1704 × 10^−242^ 7.3796 × 10^−275^ 1.5914 × 10^−36^ 1.0658 × 10^−25^ 2.9972 × 10^3^ 2.3919 × 10^−141^	2.5286 × 10^−72^ 1.0432 × 10^−76^ 0 0 1.1253 × 10^−35^ 5.3651 × 10^−25^ 1.9594 × 10^04^ 1.2551 × 10^−140^	*F*_14_	HBA HBA1 HBA2 GOHBA BKA ECO GOOSE NRBO	1.9601 1.4506 3.7002 3.3275 1.0179 1.3150 10.788 3.6973	2.4640 1.6106 3.9934 3.8655 0.14058 0.88036 5.9670 4.1621
*F*_3_	HBA HBA1 HBA2 GOHBA BKA ECO GOOSE NRBO	1.8839 × 10^−95^ 1.1811 × 10E^−121^ 0 0 6.2076 × 10^−82^ 5.0568 × 10^−52^ 1.0715 × 10^2^ 1.7675 × 10^−262^	9.4665 × 10^−95^ 6.3943 × 10^−121^ 0 0 3.1551 × 10^−81^ 2.4078 × 10^−51^ 67.106 0	*F*_15_	HBA HBA1 HBA2 GOHBA BKA ECO GOOSE NRBO	4.9888 × 10^−3^ 5.8892 × 10^−3^ 3.0749 × 10^−4^ 3.0782 × 10^−4^ 2.0645 × 10^−3^ 1.4895E × 10^−3^ 7.6863 × 10^−3^ 4.0657 × 10^−3^	8.6964 × 10^-3^ 1.1639 × 10^-2^ 1.0135 × 10^-10^ 1.3621 × 10^-6^ 5.6323 × 10^-3^ 3.9106 × 10^-3^ 1.7056 × 10^-2^ 7.7180 × 10^-3^
*F*_4_	HBA HBA1 HBA2 GOHBA BKA ECO GOOSE NRBO	1.3975 × 10^−57^ 1.5153 × 10^−65^ 5.6896 × 10^−239^ 3.7000 × 10^−268^ 1.9947 × 10^−44^ 7.0883 × 10^24^ 24.065 1.1857 × 10^−138^	3.5954E−57 3.5655E−65 0 0 1.0643 × 10^−43^ 4.5737 × 10^−23^ 6.4515 7.2777 × 10^−138^	*F*_16_	HBA HBA1 HBA2 GOHBA BKA ECO GOOSE NRBO	−1.0316 −1.0316 −1.0316 −1.0316 −1.0316 −1.0316 −1.0316 −1.0316	3.0917 × 10^-16^ 3.2349 × 10^-16^ 3.2812 × 10^-16^ 3.5888 × 10^-16^ 3.7532 × 10^-16^ 3.5110 × 10^-10^ 2.7862 × 10^-7^ 3.9746 × 10^-16^
*F*_5_	HBA HBA1 HBA2 GOHBA BKA ECO GOOSE NRBO	24.006 25.336 23.477 24.851 27.655 27.430 338.14 27.824	0.71976 0.70241 0.47340 0.53801 0.94309 0.47233 5.5200 × 10^2^ 0.75809	*F*_17_	HBA HBA1 HBA2 GOHBA BKA ECO GOOSE NRBO	0.39789 0.39789 0.39789 0.39789 0.39789 0.39789 0.39789 0.39789	3.0917 × 10^-16^ 3.2349 × 10^-16^ 3.2812 × 10^-16^ 3.5888 × 10^-16^ 3.7532 × 10^-16^ 3.5110 × 10^-10^ 2.7862 × 10^-7^ 3.9746 × 10^-16^
*F*_6_	HBA HBA1 HBA2 GOHBA BKA ECO GOOSE NRBO	2.4071 × 10^−2^ 0.11606 5.4509 × 10^−6^ 2.4749 × 10^−4^ 2.2955 5.9312 × 10^−3^ 5.8685 × 10^−2^ 2.9545	7.2801 × 10^−2^ 0.15916 7.5596 × 10^−6^ 1.3729 × 10^−4^ 1.5747 6.8283 × 10^−3^ 2.1420 × 10^−2^ 0.43331	*F*_18_	HBA HBA1 HBA2 GOHBA BKA ECO GOOSE NRBO	3.0000 5.7000 3.0000 4.6200 3.0000 3.0000 4.6200 3.0000	1.8485 × 10^-15^ 12.499 1.7690 × 10^-15^ 6.4772 1.8485 × 10^-15^ 7.5191 × 10^-14^ 11.455 3.3020 × 10^-15^
*F*_7_	HBA HBA1 HBA2 GOHBA BKA ECO GOOSE NRBO	3.4028 × 10^−4^ 3.8793 × 10^−4^ 1.4679 × 10^−4^ 1.2001 × 10^−4^ 3.6102 × 10^−4^ 2.6341 × 10^-4^ 0.28906 2.9395 × 10^−4^	2.6880 × 10^−4^ 2.7480 × 10^−4^ 1.0950 × 10^−4^ 1.1524 × 10^−4^ 3.0664 × 10^−4^ 2.2036 × 10^−4^ 0.12683 2.5676 × 10^−04^	*F*_19_	HBA HBA1 HBA2 GOHBA BKA ECO GOOSE NRBO	−3.8618 −3.8612 −3.8623 −3.8623 −3.8628 −3.8628 −3.8627 −3.8628	2.5872 × 10^-3^ 3.1846 × 10^-3^ 1.8908 × 10^-03^ 1.8908 × 10^-03^ 1.0031 × 10^-15^ 2.7858 × 10^-12^ 4.2399 × 10^-05^ 1.0327 × 10^-15^
*F*_8_	HBA HBA1 HBA2 GOHBA BKA ECO GOOSE NRBO	−8.8728 × 10^3^ −8.8750 × 10^3^ −1.1355 × 10^4^ −1.1591 × 10^4^ −8.6730 × 10^3^ −1.1312 × 10^4^ −7.0389 × 10^3^ −4.8415 × 10^3^	9.6921 × 10^2^ 9.2706 × 10^2^ 9.7941 × 10^2^ 7.0580 × 10^2^ 1.7590 × 10^3^ 7.9397 × 10^2^ 5.8050 × 10^2^ 7.0982 × 10^2^	*F*_20_	HBA HBA1 HBA2 GOHBA BKA ECO GOOSE NRBO	−3.2660 −3.2475 −3.2341 −3.2325 −3.2951 −3.2483 −3.1988 −3.2342	7.4959 × 10^-2^ 0.10054 7.7610 × 10^-2^ 7.8819 × 10^-2^ 5.3324 × 10^-2^ 5.8297 × 10^-2^ 3.3200 × 10^-2^ 8.3160 × 10^-2^
*F*_9_	HBA HBA1 HBA2 GOHBA BKA ECO GOOSE NRBO	0 0 0 0 0 0 1.5466 × 10^2^ 0	0 0 0 0 0 0 37.430 0	*F*_21_	HBA HBA1 HBA2 GOHBA BKA ECO GOOSE NRBO	−8.8796 −9.3659 −10.153 −10.153^1^ −10.003 −9.4977 −4.9875 −8.7948	2.9636 2.3962 3.5436 × 10^-15^ 3.7725 × 10^-15^ 1.0639 2.0217 2.9565 2.2724
*F*_10_	HBA HBA1 HBA2 GOHBA BKA ECO GOOSE NRBO	1.1944 1.0086 × 10^−10^ 4.4409 × 10^−16^ 0 4.4409 × 10^−16^ 4.4409 × 10^−16^ 16.703 4.4409 × 10^−16^	4.7756 6.2654 × 10^−10^ 0 0 0 0 5.8009 0	*F*_22_	HBA HBA1 HBA2 GOHBA BKA ECO GOOSE NRBO	−9.6398 −9.2391 −10.403 −10.403 −10.117 −9.3778 −6.9863 −9.1301	2.3204 2.7069 2.0142 × 10^-15^ 1.1349 × 10^-15^ 1.4202 2.4018 3.5595 2.1995
*F*_1_ *F*_11_	HBA HBA1 HBA2 GOHBA BKA ECO GOOSE NRBO	0 0 0 0 0 0 2.0920 × 10^2^ 0	0 0 0 0 0 0 37.885 0	*F*_23_	HBA HBA1 HBA2 GOHBA BKA ECO GOOSE NRBO	−9.0399 −9.2326 −10.536 −10.536 −10.400 −8.0526 −5.8102 −8.7555	3.0454 2.8235 2.4074 × 10^-15^ 2.6250 × 10^-15^ 0.94744 3.5864 3.7933 2.6778
*F*_12_	HBA HBA1 HBA2 GOHBA BKA ECO GOOSE NRBO	1.4532 × 10^−4^ 4.5309 × 10^−3^ 1.0044 × 10^−6^ 3.0860 × 10^−5^ 0.13958 2.7357 × 10^−4^ 5.5369 0.26140	9.4521 × 10^−4^ 1.5462 × 10^-2^ 1.0240 × 10^−6^ 2.1128 × 10^−5^ 0.21776 7.7691 × 10^−4^ 1.9790 8.2184 × 10^−2^				

**Table 4:** Performance ratings of GOHBA and other optimization algorithms.

Functions		HBA	HBA1	HBA2	GOHBA
*F*_3_	Mean Std Rank	1.1649 × 10^−72^ 2.5286 × 10^−72^ 5	3.1952 × 10^−77^ 1.0423 × 10^−76^ 4	5.1704 × 10^−242^ 0 2	7.3796 × 10^−275^ 0 1
*F*_4_	Mean Std Rank	1.3975 × 10^−57^ 3.5954 × 10^−57^ 5	1.5153 × 10^−65^ 3.5655 × 10^−65^ 4	5.6896 × 10^−239^ 0 2	3.7007 × 10^−268^ 0 1
*F*_7_	Mean Std Rank	3.4028 × 10^−4^ 2.6880 × 10^−4^ 5	3.8793 × 10^−4^ 2.7480 × 10^−4^ 7	1.4679 × 10^−4^ 1.0950 × 10^−4^ 2	1.2001 × 10^−4^ 1.1524 × 10^−4^ 1
*F*_10_	Mean Std Rank	1.1944 4.77567 7	1.0086 × 10^−10^ 6.2654 × 10^−10^ 6	4.4409 × 10^−16^ 0 3	4.4409 × 10^−16^ 0 3
*F*_11_	Mean Std Rank	0 0 4	0 0 4	0 0 4	0 0 4
*F*_13_	Mean Std Rank	0.50283 0.35069 5	0.54545 0.33952 6	6.3470 × 10^−3^ 1.0825 × 10^−2^ 1	1.0836 × 10^−2^ 2.3835 × 10^−2^ 2
*F*_17_	Mean Std Rank	0.39789 0 3	0.39789 0 3	0.39789 0 3	0.39789 0 3
*F_21_*	Mean Std Rank	−8.8796 2.9636 6	−9.3659 2.3962 5	−10.153 3.5436 × 10^−15^ 1	−10.153 3.7725 × 10^−15^ 2
*F*_22_	Mean Std Rank	−9.6398 2.3204 4	−9.2391 2.7069 6	−10.403 2.0142 × 10^−15^ 2	−10.403 1.1349 × 10^−15^ 1
*F*_23_	Mean Std Rank	−9.0399 3.0454 5	−9.2326 2.8235 4	−10.536 2.4074 × 10^−15^ 1	−10.536 2.6250 × 10^−15^ 2
Rank-Count		49	49	21	20
Ave-Rank		4.9	4.9	2.1	2.0
Overall-Rank		5.5	5.5	2	1
Functions		BKA	ECO	GOOSE	NRBO
*F*_2_	Mean Std Rank	1.5914 × 10^−36^ 1.1253 × 10^−35^ 6	1.0658 × 10^−25^ 5.3651 × 10^−25^ 7	2.9972 × 10^3^ 1.9594 × 10^4^ 8	2.3919 × 10^−141^ 1.2551 × 10^−140^ 3
*F*_4_	Mean Std Rank	1.9947 × 10^−44^ 1.0643 × 10^−43^ 6	7.0883 × 10^−24^ 4.5737 × 10^−23^ 7	24.065 6.4515 8	1.1857 × 10^−138^ 7.2777 × 10^−138^ 3
*F*_7_	Mean Std Rank	3.6102 × 10^−4^ 3.0664 × 10^−4^ 6	2.6341 × 10^−4^ 2.2036 × 10^−4^ 3	0.28906 0.12683 8	2.9395 × 10^−4^ 2.5676 × 10^−4^ 4
*F*_10_	Mean Std Rank	4.4409 × 10^−16^ 0 3	4.4409 × 10^−16^ 0 3	16.703 5.8009 × 10^0^ 8	4.4409 × 10⁻¹⁶ 0.0000 × 10⁰ 3
*F*_11_	Mean Std Rank	0 0 4	0 0 4	2.0920 × 10^2^ 37.885 8	0 0 4
*F*_13_	Mean Std Rank	1.8507 0.48175 7	0.29398 0.81989 4	4.9868 × 10^−2^ 2.7722 × 10^−2^ 3	2.4079 0.42596 8
*F*_17_	Mean Std Rank	3.6102 × 10^−4^ 3.0664 × 10^−4^ 6	0.39789 1.8099 × 10^−7^ 8	0.39789 9.2929 × 10^−8^ 7	0.39789 0 3
*F*_21_	Mean Std Rank	−10.003 1.0639 3	−9.4977 2.0217 4	−4.9875 2.9565 8	−8.7948 2.2724 7
*F*_22_	Mean Std Rank	−10.117 1.4202 3	−9.3778 2.40180 5	−6.9863 3.5595 8	−9.1301 2.1995 7
*F*_23_	Mean Std Rank	−10.400 0.94744 3	−8.0526 3.5864 7	−5.8102 3.7933 8	−8.7555 2.6778 6
Rank-Count		47	52	74	48
Ave-Rank		4.7	5.2	7.4	4.8
Overall-Rank		3	7	8	4

**Table 5:** Wilcoxon signed-rank for GOHBA and its comparison algorithm.

Functions	HBA vs. GOHBA		HBA1 vs. GOHBA		HBA2 vs. GOHBA		BKA vs. GOHBA	
	*p*	*h*	*p*	*h*	*p*	*h*	*p*	*h*
*F*_1_	3.3110 × 10^−20^	1	3.3110 × 10^−20^	1	NaN	0	3.3110 × 10^−20^	1
*F*_2_	7.0660 × 10^−18^	1	7.0660 × 10^−18^	1	7.0660 × 10−18	1	7.0660 × 10^−18^	1
*F*_3_	3.3110 × 10^−20^	1	3.3110 × 10^−20^	1	NaN	0	3.3110 × 10^−20^	1
*F*_4_	7.0660 × 10^−18^	1	7.0660 × 10^−18^	1	7.0660 × 10^−18^	1	7.0660 × 10^−18^	1
*F*_5_	3.1180 × 10^−9^	1	4.0380 × 10^−5^	1	9.5300 × 10^−17^	1	1.9520 × 10^−17^	1
*F*_6_	5.2790 × 10^−6^	1	9.5400 × 10^−18^	1	7.0660 × 10^−18^	1	7.0660 × 10^−18^	1
*F*_7_	1.5240 × 10^−7^	1	4.5400 × 10^−9^	1	0.10900	0	3.6920 × 10^−7^	1
*F*_8_	4.2060 × 10^−17^	1	1.5390 × 10^−17^	1	0.31580	0	8.8640 × 10^−16^	1
*F*_9_	NaN	0	NaN	0	NaN	0	NaN	0
*F*_10_	8.2230 × 10^−2^	0	0.15940	0	NaN	0	NaN	0
*F*_11_	NaN	0	NaN	0	NaN	0	NaN	0
*F*_12_	3.2870 × 10^−8^	1	2.7840 × 10^−17^	1	7.0660 × 10^−18^	1	7.0660 × 10^−18^	1
*F*_13_	5.0380 × 10^−16^	1	9.5400 × 10^−18^	1	2.4160 × 10^−5^	1	7.0660 × 10^−18^	1
*F*_14_	0.14290	0	1.2920 × 10^−2^	1	0.51920	0	6.3950 × 10^−4^	1
*F*_15_	0.49270	0	1.8870 × 10^−5^	1	1.5440 × 10^−10^	1	0.45030	0
*F*_16_	2.6330 × 10^−2^	1	0.10970	0	0..6270	0	0.42500	0
*F*_17_	NaN	0	NaN	0	NaN	0	0.15940	0
*F*_18_	0.64770	0	0.98320	0	0.76590	0	0.26960	0
*F*_19_	0.71350	0	3.1040 × 10^−2^	1	0.94920	0	2.6060 × 10^−3^	1
*F*_20_	1.3870 × 10^−2^	1	0.30880	0	0.34250	0	0.47070	0
*F*_21_	1.8480 × 10^−2^	1	7.0320 × 10^−7^	1	0.91390	0	3.8370 × 10^−18^	1
*F*_22_	9.2660 × 10^−2^	0	2.9130 × 10^−5^	1	7.0470 × 10^−2^	0	1.5940 × 10^−18^	1
*F*_23_	2.0950 × 10^−6^	1	5.3930 × 10^−8^	1	0.36810	0	3.6880 × 10^−18^	1
**Functions**	**ECO vs. GOHBA**		**GOOSE vs. GOHBA**			**NRBO vs. GOHBA**		
	***p***	***h***	***p***		***h***	***p***	***h***	
*F*_1_	3.3110 × 10^−20^	1	3.3110 × 10^−20^		1	3.3110 × 10^-20^	1	
*F*_2_	7.0660 × 10^-18^	1	7.0660 × 10^-18^		1	7.0660 × 10^-18^	1	
*F*_3_	3.3110 × 10^-20^	1	3.3110 × 10^-20^		0	3.3110 × 10^-20^	1	
*F*_4_	7.0660 × 10^-18^	1	7.0660 × 10^-18^		1	7.0660 × 10^-18^	1	
*F*_5_	1.0750 × 10^-17^	1	7.0660 × 10^−18^		1	8.4620 × 10^−18^	1	
*F*_6_	6.3190 × 10^−16^	1	7.0660 × 10^−18^		1	7.0660 × 10^−18^	1	
*F*_7_	1.4760 × 10^−4^	1	7.0660 × 10^−18^		1	3.2740 × 10^−5^	1	
*F*_8_	0.11360	1	7.0660 × 10^−18^		1	7.0660 × 10^−18^	1	
*F*_9_	NaN	0	3.3110 × 10^−20^		1	NaN	0	
*F*_10_	NaN	0	3.3110 × 10^−20^		1	NaN	0	
*F*_11_	NaN	0	3.3110 × 10^−20^		1	NaN	0	
*F*_12_	7.0660 × 10^−18^	1	7.0660 × 10^−18^		1	7.0660 × 10^−18^	1	
*F*_13_	7.0660 × 10^−18^	1	2.7980 × 10^−14^		1	7.0660 × 10^−18^	1	
*F*_14_	6.3950 × 10^−4^	0	1.6950 × 10^−11^		1	0.11500	0	
*F*_15_	0.45030	1	7.0660 × 10^−18^		1	2.7920 × 10^−11^	1	
*F*_16_	0.42500	1	2.0940 × 10^−18^		1	8.6570 × 10^−2^	0	
*F*_17_	0.15940	1	3.3110 × 10^−20^		1	NaN	0	
*F*_18_	0.26960	1	2.6330 × 10^−14^		1	3.4860 × 10^−2^	1	
*F*_19_	2.6060 × 10^−3^	1	1.3570 × 10^−14^		1	1.0770 × 10^−2^	1	
*F*_20_	0.47070	0	6.4630 × 10^−6^		1	6.3850 × 10^−2^	0	
*F*_21_	3.8370 × 10^−18^	1	2.7650 × 10^−18^		1	2.7650 × 10^−18^	1	
*F*_22_	1.5940 × 10^−18^	1	1.5940 × 10^−18^		1	1.6440 × 10^−18^	1	
*F_23_*	3.6880 × 10^−18^	1	3.0830 × 10^−18^		1	3.0830 × 10^−18^	1	

**Table 6:** Comparison of the results of robot gripper design optimization problem.

Algorithm	Best-Pos							Best-Score
	a	b	c	e	f	l	*δ*	
HBA	1.5000 × 10^2^	1.5000 × 10^2^	2.0000 × 10^2^	0	10.000	1.0000 × 10^2^	1.5978	4.2893
HBA1	1.5000 × 10^2^	95.763	2.0000 × 10^2^	50.000	1.5000 × 102	1.5059 × 10^2^	3.1399	4.1529
HBA2	1.0238 × 10^2^	10.000	1.7590 × 10^2^	0	10.000	1.0000 × 10^2^	1.0000	7.4389 × 10^−16^
GOHBA	1.0000 × 10^2^	38.197	2.0000 × 10^2^	0	10.000	1.0000 × 10^2^	1.5610	7.2741 × 10^−17^
BKA	99.870	38.066	1.7466 × 10^2^	0	32.737	1.0000 × 10^2^	1.5215	8.4241 × 10^−17^
ECO	1.5000 × 10^2^	1.0825 × 10^2^	1.5296 × 10^2^	34.726	1.3030 × 10^2^	1.6653 × 10^2^	3.1400	5.4861
GOOSE	1.2231 × 10^2^	1.1863 × 10^2^	1.9356 × 10^2^	16.159	58.996	1.7274 × 10^2^	2.4542	80.715
NRBO	1.4884 × 10^2^	1.4454 × 10^2^	1.8424 × 10^2^	0.56062	12.874	1.6113 × 10^2^	1.7935	3.8083

**Table 7:** Comparison of the results of speed reducer design problem.

Algorithm	Best-Pos							Best-Score
	*b*	*m*	*p*	*l1*	*l2*	*d1*	*d2*	
HBA	3.50000	0.70000	17.000	7.3000	7.7153	3.3502	5.2867	2.9945 × 10^3^
HBA1	3.5047	0.70000	17.000	7.3000	7.7153	3.3502	5.2867	2.9963 × 10^3^
HBA2	3.5000	0.70000	17.000	7.3000	7.7155	3.3502	5.2867	2.9945 × 10^3^
GOHBA	3.5000	0.70000	17.000	7.3000	7.7154	3.3502	5.2867	2.9945 × 10^3^
BKA	3.5000	0.70000	17.000	7.9660	7.9278	3.3515	5.2868	3.0054 × 10^3^
ECO	3.5026	0.70000	17.000	8.1823	7.7605	3.3549	5.2867	3.0059 × 10^3^
GOOSE	3.5030	0.70000	17.000	7.4587	8.3000	3.3553	5.2874	3.0117 × 10^3^
NRBO	3.5000	0.70000	17.000	7.3000	8.2906	3.3502	5.4968	3.1419 × 10^3^

**Table 8:** Comparison of GOHBA with the other 7 algorithms.

Algorithm	Map			
		MAP1	MAP2	MAP3
BKA	Mean	32.142	47.456	65.6981
	Std	7.2900 × 10^−15^	2.1870 × 10^−14^	0
ECO	Mean	32.084	47.456	65.698
	Std	0.26197	2.1870 × 10^−14^	0
GOHBA	Mean	32.025	47.456	65.698
	Std	0.30645	2.1870 × 10^−14^	0
GOOSE	Mean	32.084	47.56	65.6981
	Std	0.18030	2.1870 × 10^−14^	0
HBA	Mean	32.142	47.456	65.698
	Std	7.2900 × 10^−15^	2.1870 × 10^−14^	0
HBA1	Mean	32.054	47.456	65.698
	Std	0.28666	2.1870 × 10^−14^	0
HBA2	Mean	32.084	47.456	65.6981
	Std	0.18030	2.1870 × 10^−14^	0
NRBO	Mean	32.142	47.456	65.698
	Std	7.2900 × 10^−15^	2.1870 × 10^−14^	0

## Data Availability

The data generated from the analysis in this study can be found in this article. This study does not report the original code, which is available for academic purposes from the lead contact. Any additional information required to reanalyze the data reported in this paper is available from the lead contact upon request.

## References

[1] Li H., Fang C., Lin Z. (2020). Accelerated first-order optimization algorithms for machine learning. Proc. IEEE.

[2] Zhao D., Liu L., Yu F., Heidari A.A., Wang M., Oliva D., Muhammad K., Chen H. (2021). Ant colony optimization with horizontal and vertical crossover search: Fundamental visions for multi-threshold image segmentation. Expert Syst. Appl..

[3] Abdel-Basset M., Mohamed R., Zidan M., Jameel M., Abouhawwash M. (2023). Mantis Search Algorithm: A novel bio-inspired algorithm for global optimization and engineering design problems. Comput. Methods Appl. Mech. Eng..

[4] Barzilai J., Borwein J.M. (1988). Two-point step size gradient methods. IMA J. Numer. Anal..

[5] Borgwardt K.H. (1982). The average number of pivot steps required by the simplex method is polynomial. Math. Program..

[6] Agrawal P., Abutarboush H.F., Ganesh T., Mohamed A.W. (2021). Metaheuristic algorithms on feature selection: A survey of one decade of research (2009–2019). IEEE Access.

[7] Akinola O.O., Ezugwu A.E., Agushaka J.O., Abu Zitar R., Abualigah L. (2022). Multiclass feature selection with metaheuristic optimization algorithms: A review. Neural Comput. Appl..

[8] Ahmed I., Alvi U.-E., Basit A., Rehan M., Hong K.-S. (2022). Multi-objective whale optimization approach for cost and emissions scheduling of thermal plants in energy hubs. Energy Rep..

[9] Kiani F., Nematzadeh S., Anka F.A., Findikli M.A. (2023). Chaotic sand cat swarm optimization. Mathematics.

[10] Wu J., Su Z. (2024). Flavoring search algorithm with applications to engineering optimization problems and robot path planning. Appl. Math. Model..

[11] Shen Y., Zhang C., Farhad S.G., Mirjalili S. (2023). An improved whale optimization algorithm based on multi-population evolution for global optimization and engineering design problems. Expert Syst. Appl..

[12] Amiri M.H., Hashjin N.M., Montazeri M., Mirjalili S., Khodadadi N. (2024). Hippopotamus optimization algorithm: A novel nature-inspired optimization algorithm. Sci. Rep..

[13] Peraza-Vázquez H., Peña-Delgado A., Merino-Treviño M., Morales-Cepeda A.B., Sinha N. (2024). A novel metaheuristic inspired by horned lizard defense tactics. Artif. Intell. Rev..

[14] Hashim F.A., Houssein E.H., Hussain K., Mabrouk M.S., Al-Atabany W. (2022). Honey Badger Algorithm: New metaheuristic algorithm for solving optimization problems. Math. Comput. Simul..

[15] Xu Y., Zhong R., Cao Y., Zhang C., Yu J. (2025). Symbiotic mechanism-based honey badger algorithm for continuous optimization. Clust. Comput..

[16] Majumdar P., Mitra S. (2024). Enhanced honey badger algorithm based on nonlinear adaptive weight and golden sine operator. Neural Comput. Appl..

[17] Sun J., Wang L., Razmjooy N. (2023). Anterior cruciate ligament tear detection based on deep belief networks and improved honey badger algorithm. Biomed. Signal Process. Control..

[18] Jose R.A., Paulraj E.D., Rajesh P. (2024). Enhancing Steady-State power flow optimization in smart grids with a hybrid converter using GBDT-HBA technique. Expert Syst. Appl..

[19] Guo L., Xu C., Yu T., Wumaier T., Han X. (2024). Ultra-short-term wind power forecasting based on long short-term memory network with modified honey badger algorithm. Energy Rep..

[20] Düzenli T., Onay F.K., Aydemi S.B. (2022). Improved honey badger algorithms for parameter extraction in photovoltaic models. Optik.

[21] Ye Z., Zhao T., Liu C., Zhang D., Bai W. (2023). An Improved Honey Badger Algorithm through Fusing Multi-Strategies. Comput. Mater. Contin..

[22] Yang B., Zhou Y., Liu B., Li M., Duan J., Cao P., Zheng C., Jiang L., Sang Y. (2024). Optimal array layout design of wave energy converter via honey badger algorithm. Renew. Energy.

[23] Bansal A.K., Sangtani V.S., Bhukya M.N. (2024). Optimal configuration and sensitivity analysis of hybrid nanogrid for futuristic residential application using honey badger algorithm. Energy Convers. Manag..

[24] Huang P., Zhou Y., Deng W., Zhao H., Luo Q., Wei Y. (2024). Orthogonal opposition-based learning honey badger algorithm with differential evolution for global optimization and engineering design problems. Alex. Eng. J..

[25] Adegboye O.R., Feda A.K., Ishaya M.M., Agyekum E.B., Kim K.-C., Mbasso W.F., Kamel S. (2024). Antenna S-parameter optimization based on golden sine mechanism based honey badger algorithm with tent chaos. Heliyon.

[26] Fu Y., Liu D., Fu S., Chen J., He L. (2024). Enhanced Aquila optimizer based on tent chaotic mapping and new rules. Sci. Rep..

[27] Huang Y., Liu Q., Song H., Han T., Li T. (2024). CMGWO: Grey wolf optimizer for fusion cell-like P systems. Heliyon.

[28] Duan Y., Yu X. (2023). A collaboration-based hybrid GWO-SCA optimizer for engineering optimization problems. Expert Syst. Appl..

[29] Tanyildizi E., Demir G. (2017). Golden Sine Algorithm: A Novel Math-Inspired Algorithm. Adv. Electr. Comput. Eng..

[30] Bae S. (2019). Big-O Notation. JavaScript Data Structures and Algorithms.

[31] Wu L., Huang X., Cui J., Liu C., Xiao W. (2023). Modified adaptive ant colony optimization algorithm and its application for solving path planning of mobile robot. Expert Syst. Appl..

[32] Röhmel J. (1997). The permutation distribution of the Friedman test. Comput. Stat. Data Anal..

[33] Dewan I., Rao B.P. (2005). Wilcoxon-signed rank test for associated sequences. Stat. Probab. Lett..

[34] Antczak T. (2015). Exactness of penalization for exact minimax penalty function method in nonconvex programming. Appl. Math. Mech..

[35] Wang K., Guo M., Dai C., Li Z. (2022). Information-decision searching algorithm: Theory and applications for solving engineering optimization problems. Inf. Sci..

[36] Zhao W.G., Zhang Z.X., Wang L.Y. (2020). Manta ray foraging optimization: An effective bio-inspired optimizer for engineering applications. Eng. Appl. Artif. Intell..

[37] Miao C., Chen G., Yan C., Wu Y. (2021). Path planning optimization of indoor mobile robot based on adaptive ant colony algorithm. Comput. Ind. Eng..

[38] Guan Z., Ren C., Niu J., Wang P., Shang Y. (2023). Great Wall construction algorithm: A novel meta-heuristic algorithm for engineer problems. Expert Syst. Appl..

